# ZmMADS47 Regulates Zein Gene Transcription through Interaction with Opaque2

**DOI:** 10.1371/journal.pgen.1005991

**Published:** 2016-04-14

**Authors:** Zhenyi Qiao, Weiwei Qi, Qian Wang, Ya’nan Feng, Qing Yang, Nan Zhang, Shanshan Wang, Yuanping Tang, Rentao Song

**Affiliations:** 1 Shanghai Key Laboratory of Bio-Energy Crops, School of Life Sciences, Shanghai University, Shanghai, China; 2 Coordinated Crop Biology Research Center (CBRC), Beijing, China; 3 National Maize Improvement Center of China, China Agricultural University, Beijing, China; University of Minnesota, UNITED STATES

## Abstract

Zeins, the predominent storage proteins in maize endosperm, are encoded by multiple genes and gene families. However, only a few transcriptional factors for zein gene regulation have been functionally characterized. In this study, a MADS-box protein, namely ZmMADS47, was identified as an Opaque2 (O2) interacting protein via yeast two-hybrid screening. The N-terminal portion of ZmMADS47 contains a nuclear localization signal (NLS), and its C-terminal portion contains a transcriptional activation domain (AD). Interestingly, the transcriptional activation activity is blocked in its full length form, suggesting conformational regulation of the AD. Molecular and RNA-seq analyses of Zm*MADS47 RNAi* lines revealed down regulation of α-zein and 50-kD γ-zein genes. ZmMADS47 binds the CATGT motif in promoters of these zein genes, but ZmMADS47 alone is not able to transactivate the promoters. However, when both O2 and ZmMADS47 are present, the transactivation of these promoters was greatly enhanced. This enhancement was dependent on the AD function of ZmMADS47 and the interaction between ZmMADS47 and O2, but it was independent from the AD function of O2. Therefore, it appears interaction with O2 activates ZmMADS47 on zein gene promoters.

## Author Summary

A newly identified transcription factor of seed storage proteins can engage its transactivation ability after interacting with another seed storage protein transcription factor in maize.

## Introduction

In maize (*Zea mays*) kernels, starch, storage proteins and oil are the three main metabolic storage materials. Zeins are the most abundant storage proteins and are encoded by different classes of genes. According to current nomenclature, zeins are identified as α-, β-, γ- and δ-types based on difference in aqueous solubility and ability to form disulfide bands [1]. α-zeins are divided into two major classes: 19-kD α-zeins (z1A, z1B, z1D), and 22-kD α-zeins (z1C) [2]. The 14-kD zein belongs to the β type, while the 10-kD zein and 18-kD zeins belong to the δ type [3]. The other three zeins, including 16-kD, 27-kD and 50-kD proteins, all belong to γ-zein gene sub-family [4]. Due to their high levels of expression and complexity, zein gene transcriptional regulation has attracted the interest of researchers and breeders for decades.

The maize kernel-specific transcription factor (TF) Opaque2 (O2) is a classic basic leucine zipper (bZIP) protein factor that regulates zein synthesis [5]. Previous research showed that defective expression of O2 in maize kernels can result in a 50 to 70% reduction in zein content [6]. Because zeins contain no lysine, an essential amino acid, the amount of lysine is enriched in the *o2* mutant since lysine-containing non-zein proteins are increased [7]. *O2* was first cloned by transposon tagging in 1987 [8]. It recognizes many motifs in zein promoters, like the O2 box (5’-TCCACGTAGA-3’) in 22-kD α-zeins (z1C) [9]. O2 also regulates the other α-zeins (19-kD α-zeins), as well as the 10-kD γ-zein, the 14-kD β-zein, the 27-kD γ-zein and the 50-kD γ-zein also have been shown to be targets of O2; its most conserved binding motif is TGACGTGG [10]. Besides zeins, O2 also has non-zein transcriptional targets, such as pyruvate orthophosphate dikinase (PPDK) and lactoglutathione lysase [10]. Moreover, despite direct interaction with promoters of zeins, O2 can also bind chromatin modifiers, showing that the DNA methylation and histone modification states of zeins genes play a role in the O2-mediated zeins activation [11].

Previous studies revealed that O2 is not the only zein gene TF. The Prolamine-Box Binding Factor (PBF) was identified as another TF for zein gene expression. It is a Dof-type protein that binds a conserved sequence (TGTAAAG) found in most zein promoters, and was found to co-regulate 27-kD γ-zein expression [12,13]. Two O2 heterodimerizing proteins (OHP1/OHP2), which interact with O2 and PBF, were found to modulate 27-kD γ-zein expression [13–15]. Because O2, PBF and OHP1/OHP2 do not apparently regulate all zein genes, we envisioned that there might be more TFs for zein gene regulation.

In plants, TFs compose one of the largest gene categories, and many of these genes were identified as important regulators in multiple biological processes. With the development of genome sequencing technology, many TF genes were identified, belonging to MYB-(R1)R2R3, AP2/ERBP, bHLH, NAC, C2H2(Zn), HB, MADS, bZIP TF families [16–17]. The MADS-box proteins are a large TF family in eukaryotes that includes two sub-families termed SRF-like (type I) and MEF2-like (type II) [18]. Type I is represented by Arg80/SRF genes (in animals and fungi), while type II is represented by MIKC- and MEF2-types [19]. MIKC-type proteins are plant specific and often contain four functional domains, the MADS-box conserved domain (MADS-box), intervening (I), K-box domain, which is homologous to keratin (K), and the C terminal domain [20]. MIKC-type MADS-box TFs can be further subdivided into several subgroups, including the StMADS11-like subgroup [18].

In order to search for additional TFs that regulate zein genes and expand the potential regulatory network of O2, we performed yeast two-hybrid (Y2H) screens using a fragment of O2 containing the bZIP motif. The study yielded several O2-interacting proteins including ZmTaxilin [21]. Among the O2-interacting proteins is a MADS-box protein named ZmMADS47. In this study, we carried out a comprehensive functional analysis of ZmMADS47, and showed it is an important TF for zein gene expression, specifically for α-zeins and the 50-kD γ-zein gene. ZmMADS47 binds zein promoters next to O2 via a conserved CATGT motif, and its transactivation activity is modulated via protein-protein interaction with O2.

## Results

### MADS-box protein ZmMADS47 is an O2-interacting protein

**Fig 1:**
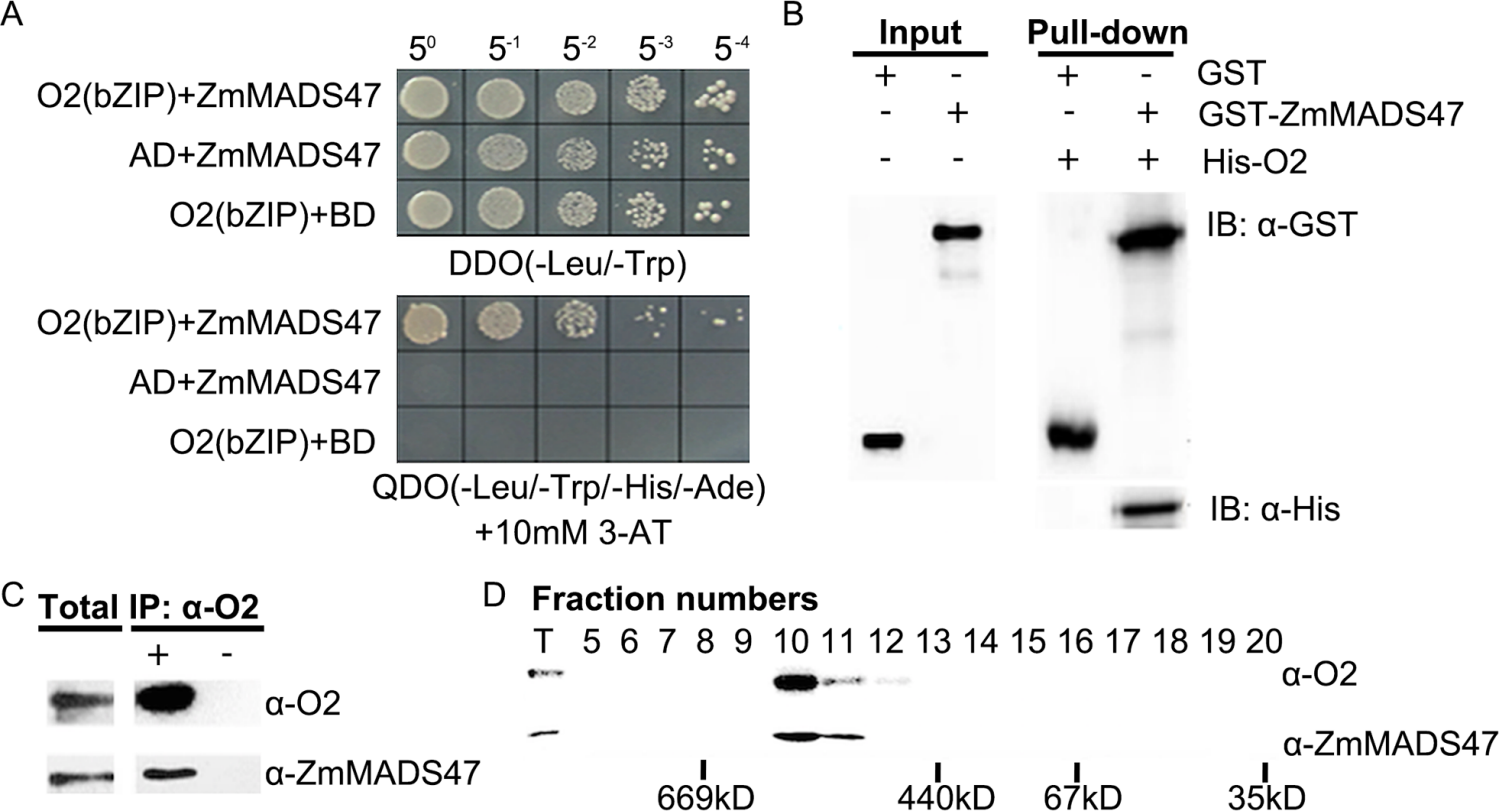
Protein interaction between ZmMADS47 and O2. **A.** Yeast two-hybrid analysis of the interaction between O2-2 (O2-bZIP) and a MADS protein. AD: GAL4 activation domain; BD: GAL4 DNA binding domain; O2 (bZIP): the O2-2 bZIP fragment that does not contain the transactivation domain and C terminal. **B.** GST pull-down analysis of ZmMADS47 and O2. The pull-down sample was detected using GST antibody (Abcam) and His antibody (Abcam). GST and GST-ZmMADS47 were used as bait and incubated with 6XHis fused O2 protein. After incubation, GST and GST-ZmMADS47 were both eluted with glutathione. IB: immunoblot. **C.** Co-immunoprecipitation assay to confirm O2-ZmMADS47 interaction. Immatured kernel extracts were incubated with O2 antibody and precipitated by Protein A Sepharose Beads (GE Healthcare). Total protein and IPed protein immunoprecipitated by O2 antibody (purified from rabbit serum) were blotted by ZmMADS47 and O2 (purified from guinea pig serum) antibodies. **D.** O2 complex seperated by gel filtration and detected by O2 and ZmMADS47 antibodies. The 500 μl immatured kernel extracts were extracted by the protocol used for coIP and injected into a Superdex 200 10/300 GL (GE Healthcare) Column. The protein eluate was collected at 0.4 ml/min speed. T: total extract.

O2 is an important transcription factor for several zein genes. It is a typical bZIP protein that can form complexes with other TFs, or itself. Y2H screening was carried out to identify proteins interacting with O2 [21]. Among them was a MADS-box protein showing high homology to OsMADS47 (these two sequences were aligned in S1 Fig) at 83% similarity; thus we named it ZmMADS47. To confirm interaction with O2, the full-length open reading frame (ORF) of *ZmMADS47* was cloned into the pGADT7-Rec vector and transformed into yeast strain AH109 with pGBKT7-*O2-2*. The resulting transformant was cultured on QDO (SD/-Leu/-Trp/-His/-Ade) plate containing 10 mM 3-AT, where it grew well; the negative control did not grow. The result indicated the full length ZmMADS47 can interact with O2 in yeast (Fig 1A).

To confirm ZmMADS47 can directly interact with O2, a pull-down assay was carried out. ZmMADS47 was fused to a glutathione S-transferase (GST) tag, expressed and purified from *E*.*coli*. GST-tagged ZmMADS47 was incubated with purified His-tagged O2 protein, while GST was used as a negative control. After a pull-down assay, the O2 protein was detected in the sample containing GST-ZmMADS47 but not the GST control using His-tag antibody (Fig 1B). This result indicated that O2 and ZmMADS47 can interact with one another *in vitro*.

To verify the interaction between O2 and ZmMADS47 *in vivo*, co-immunoprecipitation (co-IP) was performed using total protein extract from 15-days-after-pollination (DAP) maize kernels. Given that O2 and ZmMADS47 antibodies were newly produced, we first tested the specificity of these two antibodies in immature kernels (S2 Fig). Moreover, our results published before suggested that O2-specific antibody is effective in immunoprecipitation analysis [10, 21, 22]. ZmMADS47 was clearly detected among proteins pulled down by O2 antibody, but not the negative control. Therefore, ZmMADS47 might form a complex with O2 *in vivo* (Fig 1C). A gel filtration assay was carried out to test this. Extract from immature kernels (15 DAP) was filtered by molecular weight using a Superdex 200 10/300GL Column (GE Healthcare) to separate protein complexes. The eluted fractions were analyzed by SDS-PAGE and immunoblotted with O2-specific or ZmMADS47-specific antibodies (Fig 1D). Both ZmMADS47 and O2 were detected in fractions 10 and 11. This implied that O2 and ZmMADS47 might exist in a complex of about 550 kD *in vivo*.

### ZmMADS47 is a MIKC-type MADS-box protein expressed constitutively in maize

*ZmMADS47* is located on maize chromosome 1 (17,964,695–17,986,258 bp) and contains eight exons and seven introns, according to the B73 genome assembly (B73 RefGen_v2) [23]. The complete *ZmMADS47* cDNA is 702 bp and encodes a protein of 24 kD. BLASTX analysis indicated ZmMADS47 is a typical MIKC-type MADS-box TF containing three predicted functional domains (S3A Fig): a conserved MADS-box (1–73 aa) at the N-terminus predicted to be involved in DNA binding and nuclear localization; a keratin-like (K-box) (73–176 aa) in the middle portion with potential dimerization; and a non-conserved C-terminus (177–233 aa) that might function in transactivation.

To compare the relationship of ZmMADS47 to other MADS-box polypeptide sequences, a phylogenetic tree was constructed. Ten highly similar proteins were selected from maize and rice (*Oryza sativa*). According to this analysis, *ZmMADS47* belongs to the StMADS11-like MADS-box gene subfamily [24], and is most closely related to rice *OsMADS47* (S3B Fig). In maize, *ZmMADS19*, *ZmMADS26*, *ZmMADS21* and *ZmMADS22* are most closely related to *ZmMADS47*. Previous studies indicated that OsMADS47 might affect floral reversions [25], and it could negatively modulate brassinosteroid responses [26].

Various tissues were collected from maize plants to analyze the expression of *ZmMADS47* RNA by quantitative real time PCR (qRT-PCR). *ZmUBQ* (GenBank Accession Number: BT018032) was used as an internal control. This analysis showed *ZmMADS47* RNA is highly expressed in root, stalk and husk, but weakly expressed in leaf and ear (S3C Fig, left). In 15-DAP kernel, *ZmMADS47* RNA was preferentially expressed in endosperm. During kernel development, expression of *ZmMADS47* RNA rose to peak level at approximately 15 DAP and then gradually declined with kernel maturation (S3C Fig, right). A western blot using ZmMADS47-specific antibody revealed protein accumulation patterns similar to those of its RNA (S3D Fig). Additionally, the expression profile of *O2* was analysed. As expected, *O2* RNA could only be abundantly detected in endosperm (S3E Fig, left). In addition, compared with *ZmMADS47* RNA, the accumulation of *O2* transcripts rose to a peak at ~21 DAP and could not be detected until 12 DAP (S3E Fig, right). The protein expression pattern of O2 was similar to that of its transcript (S3F Fig).

### ZmMADS47 is a nuclear-localized proteins

**Fig 2:**
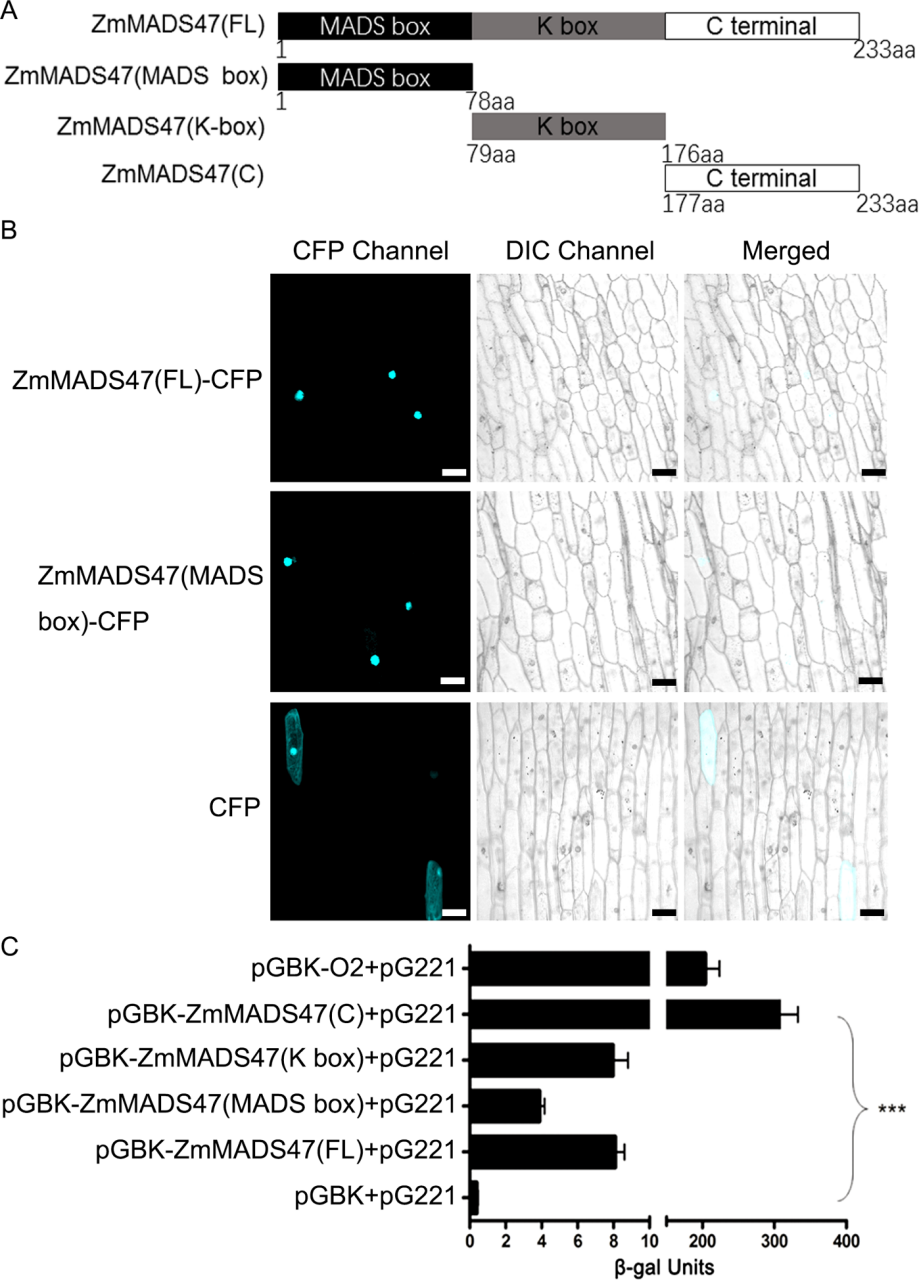
Sub-cellular localization and transactivation identification of ZmMADS47. **A.** Schematic representation of multiple truncated ZmMADS47 constructions. **B.** Fluorescence signal resulting from expression of ZmMADS47 (FL)-CFP, ZmMADS47 (MADS)-CFP and CFP alone. Signal from CFP, DIC (differential-interference microscope) and merging the two signals are shown in these panels. Bar represents 50μm. **C.** The β-galactosidase activity resulting from the TF transactivation. O2 was used as a positive control, while the pGBK-T7 vector alone was used as a negative control. Error bars represent SD (n = 3) (***P < 0.001, Student’s t test).

O2 is a bZIP TF found in endosperm nuclei [9]. In addition, N-terminal of MADS-box conserved domain in type II MADS box protein was demonstrated as nuclei localization signal in previous reaearches [27]. Taking clues from these studies, full-length ZmMADS47 [ZmMADS47(FL)] and each of its three domain segments (ZmMADS47(MADS-box), ZmMADS47 (K-box), and ZmMADS47 (C)) were cloned into an expression vector and fused with a cyan fluorescence protein (CFP, Fig 2A), in order to analyze the sub-cellular localization of ZmMADS47. After bombarding constructs into onion (*Allium cepa*) epidermal cells, the fusion proteins were detected by laser confocal microscopy. The CFP signal showed the full length ZmMADS47 and N terminal MADS-box segment were both localized to nuclei, while the CFP control was localized to both nuclei and cytosol (Fig 2B). Considering the fact that CFP alone cannot be located in nuclei specifically, we lead to a conclusion that the N-terminal MADS-box segment can be located in nuclei.

### The C-terminal portion of ZmMADS47 has transactivation activity

To investigate if ZmMADS47 has transcriptional transactivation activity, a yeast transactivation assay was carried out. The full-length ZmMADS47 cDNA and its three partial segments were cloned into the pGBK-T7 vector, and co-transformed into the EGY48 yeast strain with the pG221 vector. pG221 contains a β*-galactosidase* reporter gene with a minimum promoter that can be bound by a DNA binding domain protein expressed from the pGBK-T7 vector. The β-galactosidase assay showed that full-length ZmMADS47 has very low measurable transactivation activity—probably at background levels compared to O2, the positive control. However, the C-terminal portion of ZmMADS47 could significantly transactivate the reporter gene (Fig 2C). These results suggest that the transactivation activity by the C-terminus of ZmMADS47 was presumably prevented by conformational changes when the full-length protein was assayed.

### Transgenic RNAi lines can significantly suppress *ZmMADS47* expression

RNAi was used to generate *ZmMADS47* knockdown transgenic plants. A 300-bp fragment from the *ZmMADS47* ORF was selected to construct the pFGC-5941 RNAi vector (S4A Fig). The 16-kD zein promoter was used to drive expression substituting for the original promoter. The salient feature of this approach, as employed here, is the specific expression of endogenous non-coding RNA in endosperm that would target endogenous *ZmMADS47* RNA for degradation resulting in lower transcript levels. Five independent transgenic lines (line 3, line 6, line 7, line 8, line A) were generated by *Agrobacterium tumefaciens*-mediated immature embryo transformation. Southern blot hybridization of five independent transgenic lines showed specific transgene fragments (S4B Fig). Expression of *ZmMADS47* in the RNAi lines was examined in immature kernels by real time PCR. According to this analysis, among the five RNAi lines, line 3 and line 6 confirmed decreased levels in *ZmMADS47* transcript in both lines (65.7% and 41.2%, respectively) (S4C Fig). Moreover, western blot analysis with ZmMADS47-specific antibody indicated a marked reduction of ZmMADS47 protein in both lines (S4D Fig). Therefore, RNAi lines 3 and 6 were selected for further analysis.

### Expression of α zeins and 50-kD γ-zein is down regulated in Zm*MADS47 RNAi* lines

To explore the potential regulatory function of ZmMADS47, we performed next-generation RNA sequencing and assembled transcriptomes of three developing wild-type (WT) and RNAi line kernels. Protocols for genotyping and bioinformatics pipline used for transcriptome reconstitution are described in detail in methods and materials. We acquired ~15 million raw reads for each sample. About 10 million reads were unique and were used to calculate the relative abundance of transcripts (expressed as transcript sequence per million base pairs sequenced fragments per kilobase of exon per million fragments mapped, FPKM). After cleaning and mapping, we defined the differentially expressed genes (DEGs) as those with a fold change> 1.40 or <0.71 and P value <0.05. Using these criteria, 1071 DEGs were identified. In addition, several ZmMADS47-homologous genes, including *ZmMADS19*, *ZmMADS26*, *ZmMADS21* and *ZmMADS22* were not part of the DEG set identified, showing the specificity of the RNAi knockdown of *ZmMADS47*.

All 1071 DEGs were characterized using the Gene Ontology (GO) database (http://bioinfo.cau.edu.cn/agriGO/), but only a few of them could be annotated by GO database because most genes did not have a GO database match. They corresponded to five major GO terms: nutrient reservoir activity (GO0045735, P value = 1.37E-20), response to wounding (GO0009611, P value = 0.00392), defense response (GO0006952, P value = 0.00213), cellular carbohydrate metabolic process (GO0044262, P value = 0.00392) and serine-type endopeptidase inhibitor activity (GO0004867, P value = 0.000948) (S1 Table). Most DEGs in the nutrient reservoir activity term encoded zeins, especially *α-zeins* and the *50-kD γ-zein* (Table 1). There was 2.06 to 1.55 fold down-regulation in transcript levels of 19-kD α-zeins, 4.47 to 1.48 fold down-regulation of 22-kD α-zeins, and 1.44 fold down-regulation of 50-kD γ-zein.

**Fig 3:**
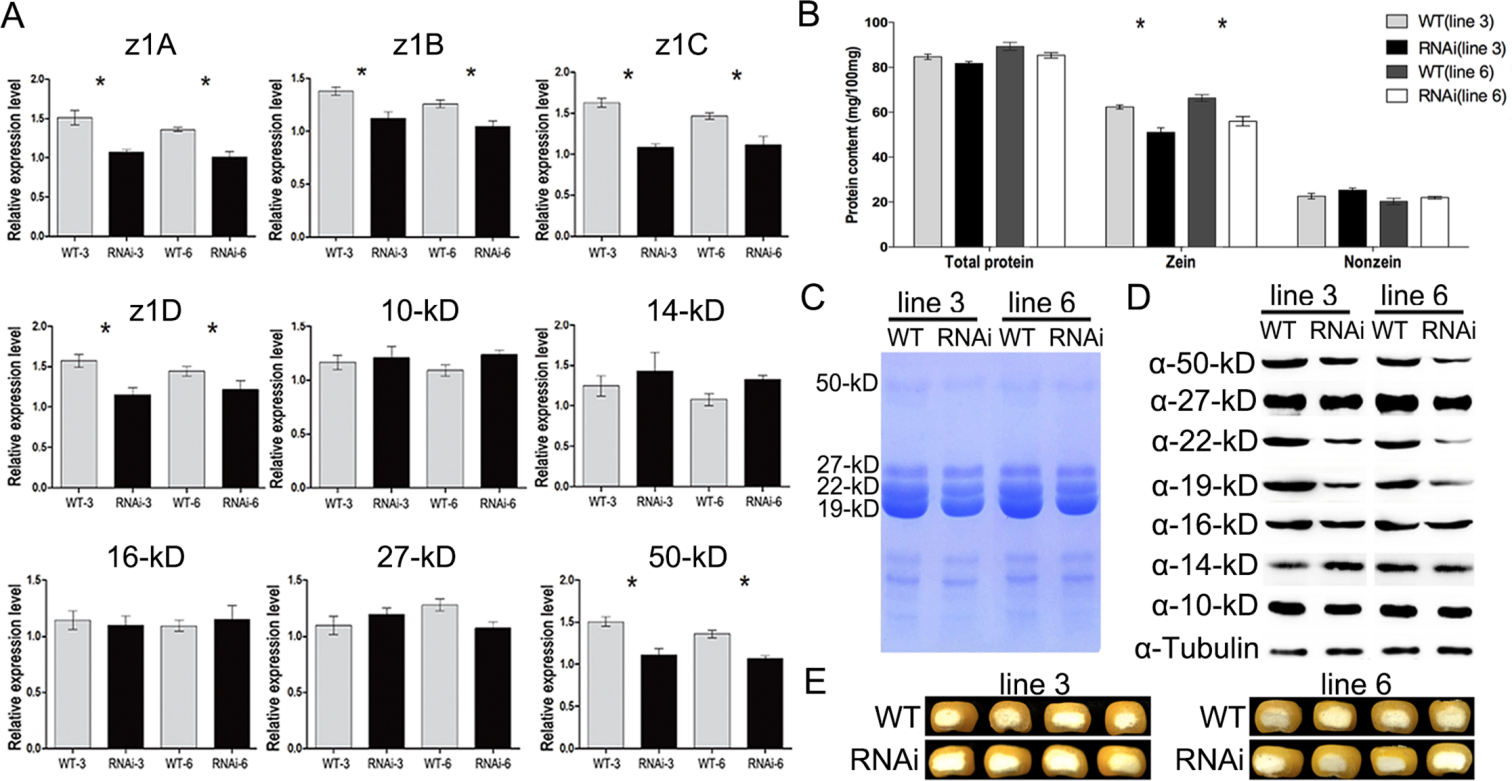
Zein content in *ZmMADS47 RNAi* transgenic lines. **A.** Real-time PCR assay to detect zein transcripts in RNAi lines 3 and 6 in. *ZmUBQ* was used as an internal control. All the assays were repeated three times. Error bars represent SD (n = 3) (*P < 0.05, Student’s t test). **B.** Comparison of zein, nonzein, and total proteins from wild type and RNAi lines 3 and 6 mature endosperm. The measurements were done on per mg of dried endosperm. Error bars represent SD (n = 3) (*P < 0.05, Student’s t test). **C.** SDS-PAGE detection of zein accumulation in RNAi lines 3 and 6. **D.** Immunoblot detection of zein proteins in RNAi lines 3and 6 with specific zein antibodies. **E.** Cross section of segregating wild type and RNAi mutant kernels from ears of lines 3 and 6 at maturity.

To corroborate the expression changes of *zeins* between WT and Zm*MADS47 RNAi* kernels, qRT-PCR was performed on all major zein gene classes. The results indicated a reduction of all four α zein classes z1A (GRMZM2G059620), z1B (AF546188.1_FG001), z1C (GRMZM2G346897) and z1D (AF546187.1_FG001) and the 50-kD γ-zein (GRMZM2G138689) (Fig 3A). z1A RNA was 33.5% down-regulated, z1B was 18.0% down-regulated, z1C was 28.5% down-regulated, z1D was 21.5% down-regulated, and the 50-kD γ-zein RNA was 24.0% down-regulated. There was no significant difference in the total protein content between wild type and Zm*MADS47 RNAi* kernels and quantitative analysis showed that zeins were significantly decreased (16.8%, Fig 3B). To examine if α zeins and the 50-kD γ-zein were decreased at the protein level, SDS-PAGE electrophoresis and western blot analyses were performed with 5 μg zeins extracted from mature kernels. The results showed a decrease of 19-kD α-zein, 22-kD α-zein and 50-kD γ-zein in lines 3 and 6 compared to wild type (Fig 3C and 3D). Despite the reduction in zein content, no apparent *opaque* which is often associated with decreased zein content, as in *o2*, was observed in RNAi mature kernels (Fig 3E).

**Table 1:** List of nutrient reservoir activity-related DEGs.

Name	Description	Fold change	P-value
AF546188.1_FG007	19-kD α-zein (19C2)	-1.55	0
GRMZM2G008913	19-kD α-zein (PMS2)	-1.71	0.0018
AF546187.1_FG007	19-kD α-zein (19D1)	-1.84	1.51E-112
AF546187.1_FG001	19-kD α-zein (az19D1)	-2.06	3.55E-193
GRMZM2G008341	19-kD α-zein (19A2)	1.63	0.027
GRMZM2G346897	22-kD α-zein (az22z4)	-1.89	0
GRMZM2G044152	22-kD α-zein (ZA1/M1)	-1.60	7.68E-72
GRMZM2G088365	22-kD α-zein (az22z5)	-4.47	0
GRMZM2G044625	22-kD α-zein (PZ22.3)	-1.48	3.16E-304
GRMZM2G160739	22-kD α-zein (B49)	-1.51	7.38E-56
GRMZM2G044152	22-kD α-zein (ZA1/M1)	-1.60	7.68E-72
GRMZM2G397687	22-kD α-zein (PZ22.1/22A1)	-1.52	0
GRMZM2G138689	50-kD γ-zein	-1.44	1.20E-133
GRMZM2G026703	Globulin-2 Precursor	-2.19	5.89E-35
GRMZM2G067919	Globulin-1 S allele Precursor (GLB1-S)(7S-like)	-1.83	2.27E-21
GRMZM2G332259	Rapid ALkalinization Factor	-4.35	0.0098
GRMZM2G089493	hypothetical protein LOC100191708	-4.00	0.020
GRMZM2G388461	unknown	-1.75	1.14E-08
AF546188.1_FG003	unknown	-1.70	0.0043
GRMZM2G346895	unknown	-1.71	9.01E-29
GRMZM2G088273	unknown	-2.71	0.00090
GRMZM2G045387	unknown	-1.77	2.94E-71

### Smaller and irregular protein bodies in Zm*MADS47 RNAi* endosperm

**Fig 4:**
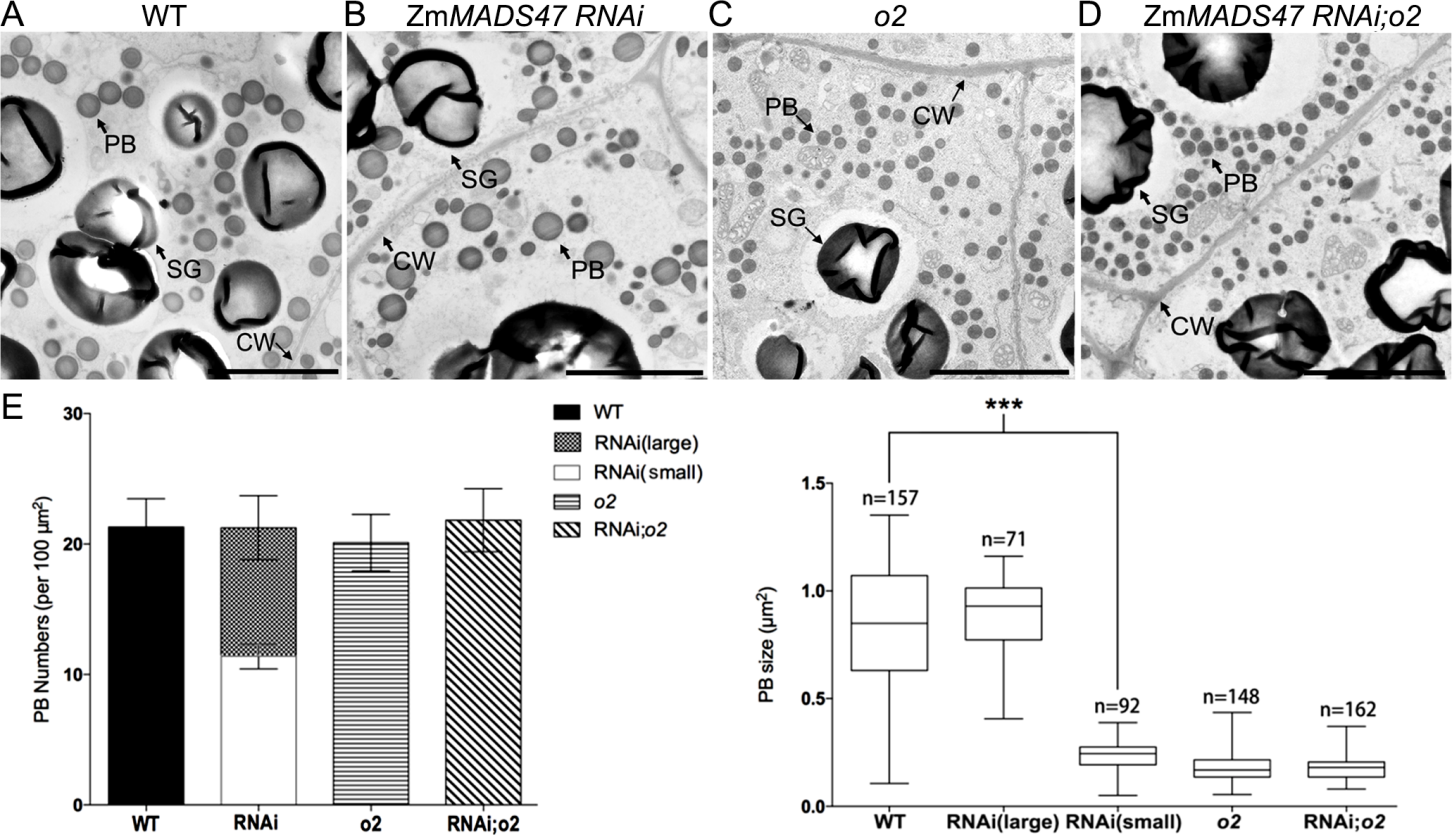
Transmission electron microscopy of wild type, Zm*MADS47 RNAi*, *o2*, and Zm*MADS47 RNAi*; *o2* starchy endosperm. **A-D.** Protein bodies in the fourth starchy endosperm cell layer at 18DAP stage were observed by transmission electron microscopy. Each genotype is labeled above the corresponding TEM image. PB: protein body; CW: cell wall; SG, starch granule. Bars = 1μm. **E.** Quantitative comparison of PB number and size in the fourth and fifth cell layer from aleurone between wild type, Zm*MADS47 RNAi*, *o2*, and Zm*MADS47 RNAi;o2* endosperm. Error bars represent SD (***P < 0.001, Student’s t test).

In maize endosperm, zeins are stored in protein bodies (PBs) [28]. To examine if there is an effect on PBs in developing *ZmMADS47* RNAi kernels, 18-DAP WT and counterpart Zm*MADS47 RNAi* kernels were prepared for transmission electron microscopy (TEM), and the morphology and size of PBs in the fourth and fifth endosperm cell layers were examined. In Zm*MADS47 RNAi* kernels, protein bodies were not uniform in size, and there were two types of protein bodies: one type was similar to those in WT; the other type was much smaller, and often irregular in shape (Fig 4A and 4B, S5A and S5B Fig). The total number of PBs was not significantly affected in Zm*MADS47 RNAi* endosperm. The number of irregular PBs was 61.9% of total PBs in RNAi endosperm. The size of normal PBs was similar between wild type and Zm*MADS47 RNAi* endosperm, while the size of small and irregularly shaped PBs in Zm*MADS47 RNAi* endosperm was only 35.6% of normal PBs (Fig 4E).

Because ZmMADS47 is an O2-interacting protein, we also constructed a Zm*MADS47 RNAi;o2* double mutant. TEM observation of *o2* and Zm*MADS47 RNAi*;*o2* at 18-DAP (Fig 4C and 4D) showed no difference in PBs between the double mutant and *o2*.

### ZmMADS47 binds CATGT motif in α zein and 50-kD γ-zein promoters *in vitro*

**Fig 5:**
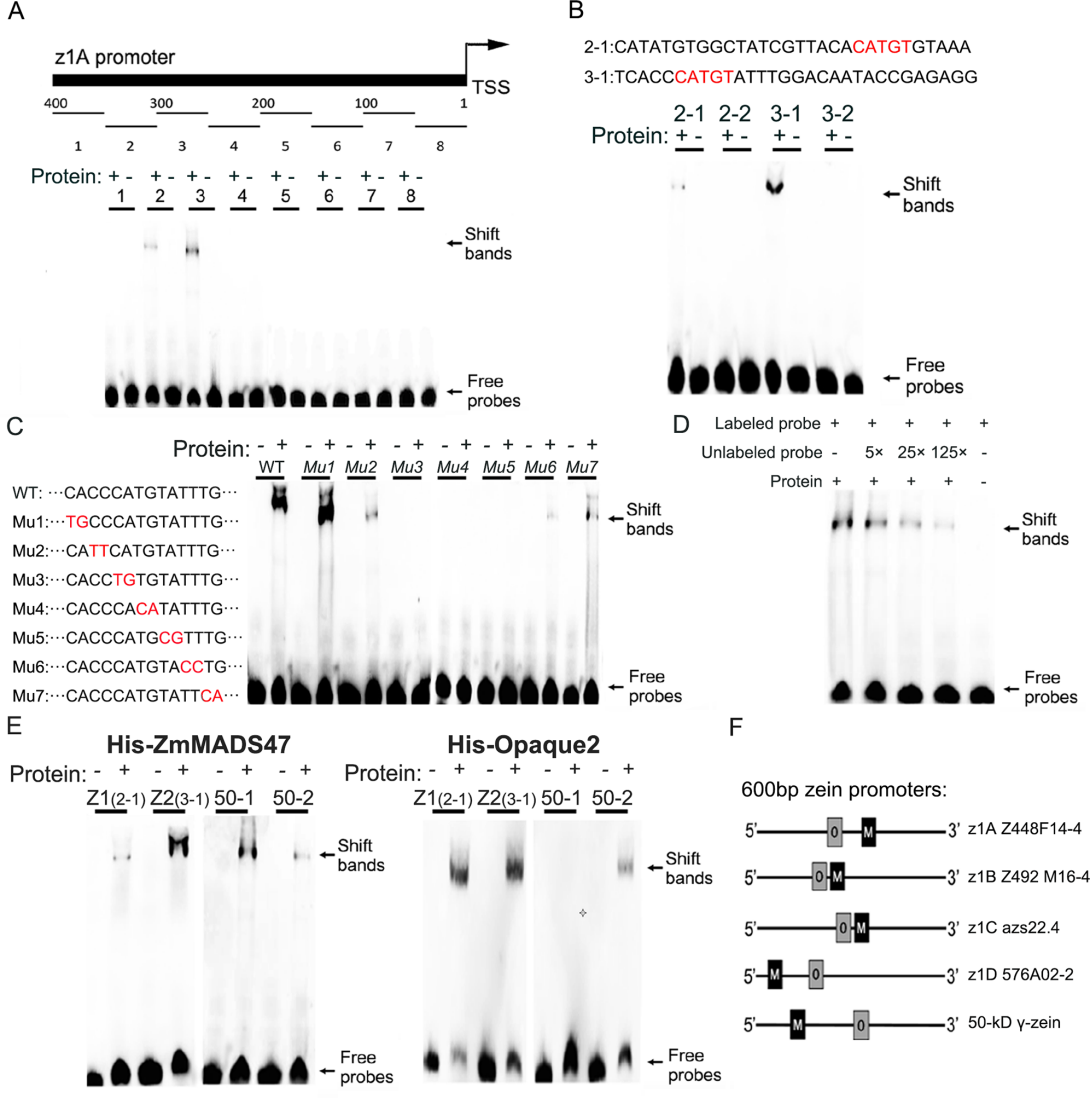
Characterization of the ZmMADS47 binding motif. **A.** Schematic representation of z1A promoter fragmentation. Black arrow points to the ZmMADS47-indused shift bands. **B.** Deletion analysis to narrow down the conserved domain recognized by ZmMADS47. The upper black arrow identifies the shifted bands due to the ZmMADS47/DNA complex. The bases highlighted by red letters represent the sharing sequences in both two fragments. **C.** Mutagenesis assay of CATGT motifs. The upper black arrow identified the shifted bands due to the ZmMADS47/DNA complex. The bases highlighted by red letters represent the mutational bases compared with wild-type bases. **D.** Effect of competitive probe on the band shift reaction. The upper black arrow identifies the shifted bands due to ZmMADS47/DNA complex. **E.** The binding ability of His-ZmMADS47 and His-O2 to CATGT motifs in the z1A promoter and 50-kD zein promoter. The experiment was performed by adding ZmMADS47 or O2 protein. The upper black arrow identifies shifted bands due to ZmMADS47/DNA complex or O2/DNA complex. **F.** Schematic representation of ZmMADS47 binding site (M) and O2 DNA binding site (O) in down-regulated zein promoters.

To examine if ZmMADS47 directly binds a unique DNA motif within the promoters of affected zein genes, an electrophoretic mobility shift assay (EMSA) was performed using purified recombinant His-ZmMADS47 protein. A 400 bp z1A promoter region, including sequences upstream of transcription start site (TSS), was divided into eight 50-bp segments, which were tested individually by EMSA with His-ZmMADS47. High-molecular-weight-shifted bands indicating protein/DNA complexes were observed for probes 2 and 3 (Fig 5A). Both probes were further divided into two segments, and tested again by EMSA (Fig 5B), and the DNA interacting region was further narrowed down to probe 2–1 and probe 3–1, respectively. Sequence analysis revealed a conserved CATGT motif presented in both segments (Fig 5B).

To verify if CATGT is the core motif recognized by ZmMADS47, probe 3–1 was subjected to point mutation analysis. This was done via a series of two bases mutations, scanning across the 14-bp fragment centered on CATGT (Fig 5C). The results indicated that once any of the five bases of the CATGT was mutated, binding ability, as indicated by the bands shift, was abolished. Therefore, CATGT is the core motif for ZmMADS47 binding. Notably, flanking bases around the CATGT core motif could also affect the affinity of DNA binding by ZmMADS47. Furthermore, we also used WT probes which already used in Fig 5C to perform competition assays, showing that ZmMADS47/DNA complexes gradually disappeared after adding unlabelled probe (Fig 5D). And the His-tag alone did not show binding activity to WT probes (S6 Fig). These results indicated that ZmMADS47 could bind the specific sequences in zein promoters.

Interestingly, we found the newly identified ZmMADS47 binding motifs in the z1A promoter, which were previously named Z1 (probe 2–1) and Z2 (probe 3–1) and which were considered as O2 binding motifs [29]. There are also two CATGTs (50–1,50–2) in ~500 bp of the 50-kD γ-zein promoter, and one of them (50–2) is also a binding motif for O2 [10]. Therefore, we performed EMSA with DNA probes containing the Z1 (TTACATGTGT) and Z2 (TCACCCATGT) motifs in the z1A α zein promoter and the 50–1 (TTGCATGTAC) and 50–2 (TGACATGTAA) motifs in the 50-kD γ-zein promoter. Shifted bands of different intensity were detected with all four probes when incubated with His-ZmMADS47 (Fig 5E). The differences in intensity suggested that the Z2 motif in the z1A promoter and the 50–1 motif in the 50-kD γ-zein promoter are preferential binding sites, with greater binding affinity for ZmMADS47. However, when His-ZmMADS47 was substituted with His-O2, the binding patterns of O2 to these motifs were affected. His-O2 bound both Z1 and Z2 motifs, but only bound the 50–2 and not the 50–1 motif (Fig 5E). The difference in the intensity of the retarded bands suggested the Z1 motif in the z1A promoter and the 50–2 motif in the 50-kD γ-zein promoter are preferential binding sites with greater binding affinity for O2. Therefore, O2 and ZmMADS47 have preferential binding sites in zein promoters (S7 Fig).

The promoters of other down-regulated zein genes in Zm*MADS47 RNAi* were also analyzed, and Fig 5F shows a schematic representation of ZmMADS47 and O2 DNA binding sites in the down-regulated α zein and 50-kD γ-zein promoters. The promoters of non-regulated zein genes in the Zm*MADS47 RNAi* line were also analyzed. O2 binding sites were also found in 10-kD, 14-kD, and 27-kD zein promoters, while none exist in the 16-kD and 18-kD zein promoters [10]. There is no ZmMADS47 binding site (CATGT) in the 10-kD and 27-kD zein promoters, and the only CATGT sequence in the 14-kD zein promoter belongs to an O2 binding site. Taking these observation into account, the sites recognized by ZmMADS47 only exist in the down-regulated zein promoters in the Zm*MADS47 RNAi*.

### ZmMADS47 promotes zein gene expression after interacting with O2

**Fig 6:**
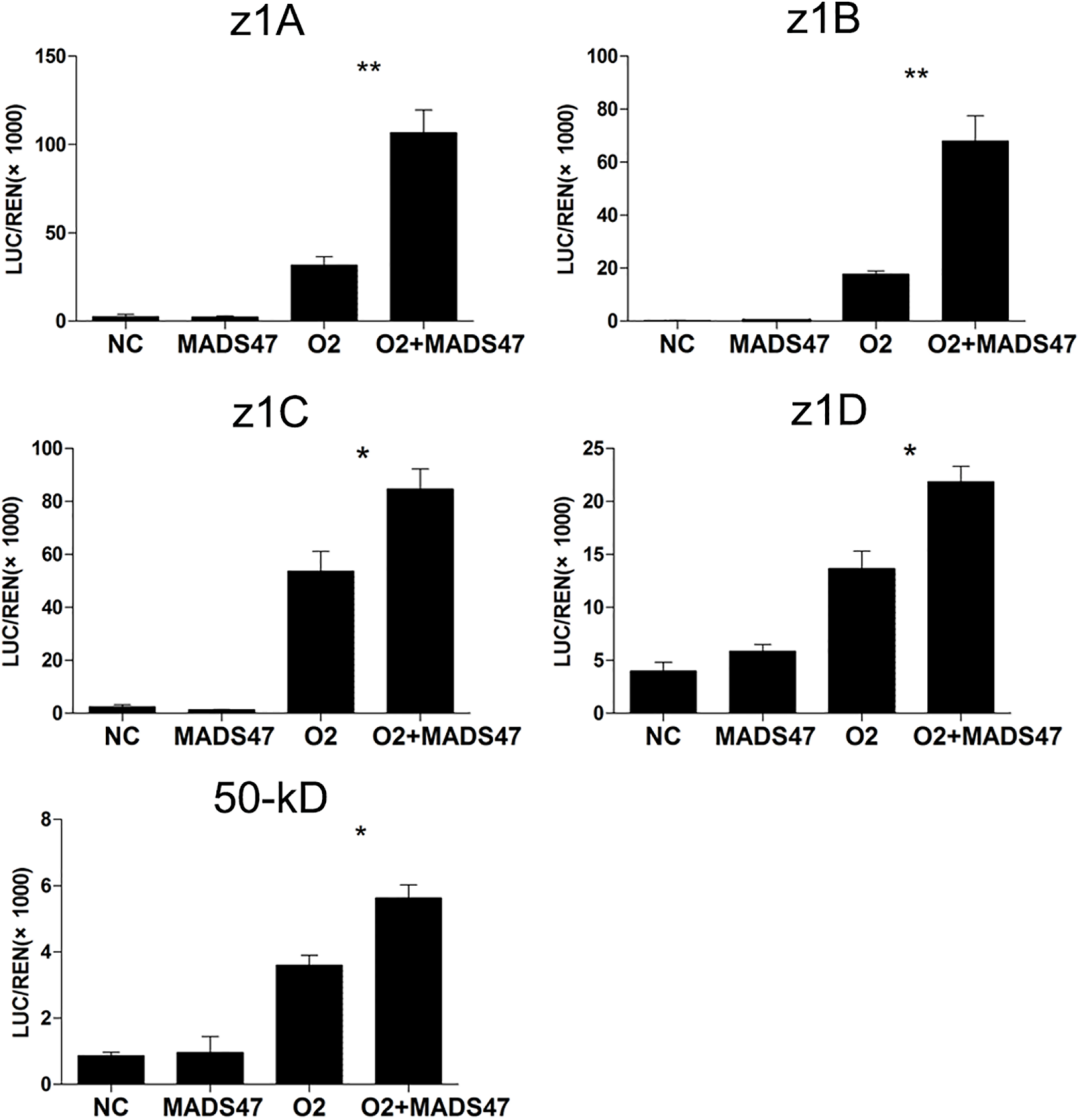
Relative transactivation of ZmMADS47 to different zein genes in onion cells. LUC/REN indicates the ratio of the firefly luciferase activity and the renilla reniformis activity. Error bars represent SD (n = 3) (*P < 0.05, **P < 0.01, ***P < 0.001, Student’s t test). NC respersent to those with the reporter genes (LUC/REN) alone.

To study the transactivation activities of ZmMADS47 and O2 on zein promoters, Luciferase (LUC) / Renilla reniformis(REN) transactivation assays were carried out in onion (*Allium cepa*) epidermal cells [30]. Expression of both O2 and ZmMADS47 was driven by the cauliflower mosaic virus (CaMV) 35S promoter, and that of the LUC reporter gene was driven by ~500 bp promoters from z1A, z1B, z1C, z1D and 50-kD zein genes. No significant transactivation of reporter gene expression was detected when ZmMADS47 was expressed alone. However, strong transactivation was detected when O2 was expressed alone (Fig 6). Interestingly, when both O2 and ZmMADS47 were expressed together, transactivation of the reporter gene was significantly higher than with O2 alone. Nevertheless, expression of other O2-targeted zeins, such as 10-kD, 14-kD, 16-kD and 27-kD zeins, was not enhanced by co-expressing ZmMADS47 (S8 Fig). These results confirmed that ZmMADS47 alone does not have transactivation activity in planta. ZmMADS47 alone can not trans-activate α zeins and 50-kD γ-zein gene promoters; however, when co-expressed with O2, it can significantly enhance the activation of these zein promoters.

### O2 releases the transactivation ability of ZmMADS47

**Fig 7:**
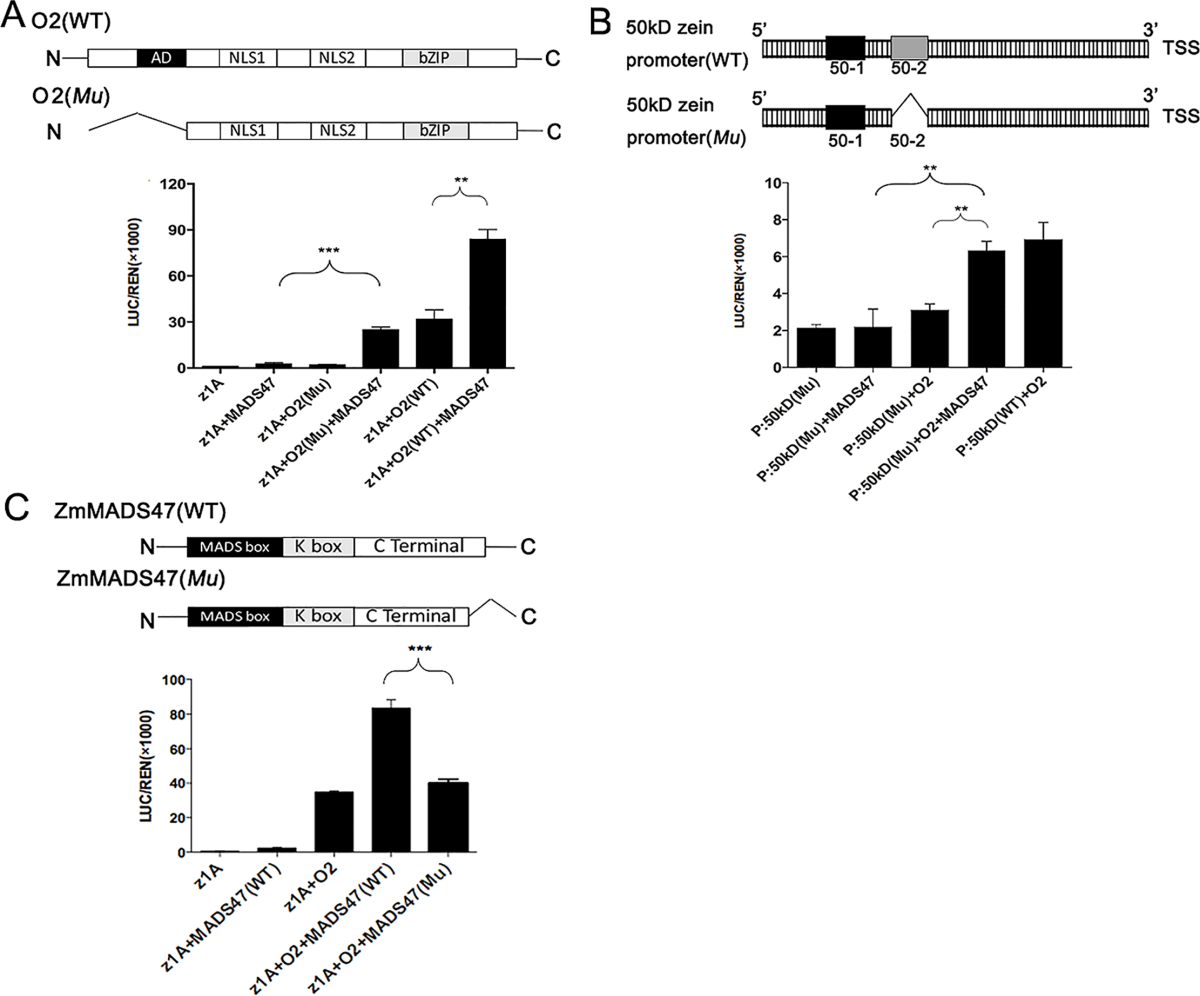
Transactivation ratio of O2 and ZmMADS47. **A.** Schematic representation of AD-truncated O2 construction. LUC/REN indicates the ratio of the firefly luciferase activity and the renilla reniformis activity. Error bars represent SD (n = 6) (*P < 0.05, **P < 0.01, ***P < 0.001, Student’s t test). **B.** Schematic representation of the 50–2 motif deletion in 50-kD γ-zein promoter. LUC/REN indicates the ratio of the firefly luciferase activity and the renilla reniformis activity. Error bars represent SD (n = 6) (*P < 0.05, **P < 0.01, ***P < 0.001, Student’s t test). **C.** Schematic representation of seven amino acid deletion at the C-terminus of ZmMADS47. LUC/REN indicate the ratio of the firefly luciferase activity and the renilla reniformis activity. Error bars represent SD (n = 6) (*P < 0.05, **P < 0.01, ***P < 0.001, Student’s t test).

To investigate the mechanism of transactivation enhancement on target genes by ZmMADS47 and O2, an activation-domain (AD)-truncated O2 (O2 (Mu)) was constructed (Fig 7A). The truncated O2 protein lacks its AD domain but still has the bZIP fragment (O2-2) that is sufficient to interact with ZmMADS47 [20]. Transactivation of zein gene expression was diminished by AD-truncated O2 as expected, yet it could still be significantly enhanced when the AD-truncated O2 and ZmMADS47 were co-expressed (Fig 7A). Arising from the truncated O2 lacking AD domain, transactivation of the zein promoter could only result from the AD of ZmMADS47. This result suggestes that the AD function of ZmMADS47 is compensated by AD-truncated O2.

To investigate whether O2 can provide the AD function of ZmMADS47 without directly binding DNA, the 50-kD γ-zein promoter was selected for further analysis. Because only the 50–2 motif was recognized by O2, while 50–1 and 50–2 motifs were both recognized by ZmMADS47, we deleted 50–2 motif in 50-kD γ-zein promoter in order to inhibit O2/DNA binding without affecting ZmMADS47/DNA binding. The 50–2 motif, the only O2-binding motif in the 50-kD γ-zein promoter, was deleted and the mutated promoter (Pro:zein (Mu)) was tested in the transactivation assay (Fig 7B). As expected, deletion of the O2 binding motif diminished O2 transactivation of the mutated 50-kD zein promoter. But surprisingly, co-expression of ZmMADS47 and O2 still can transactivate the mutated 50-kD promoter. This result suggests O2 provides AD function of ZmMADS47 even without binding the promoter.

**Fig 8:**
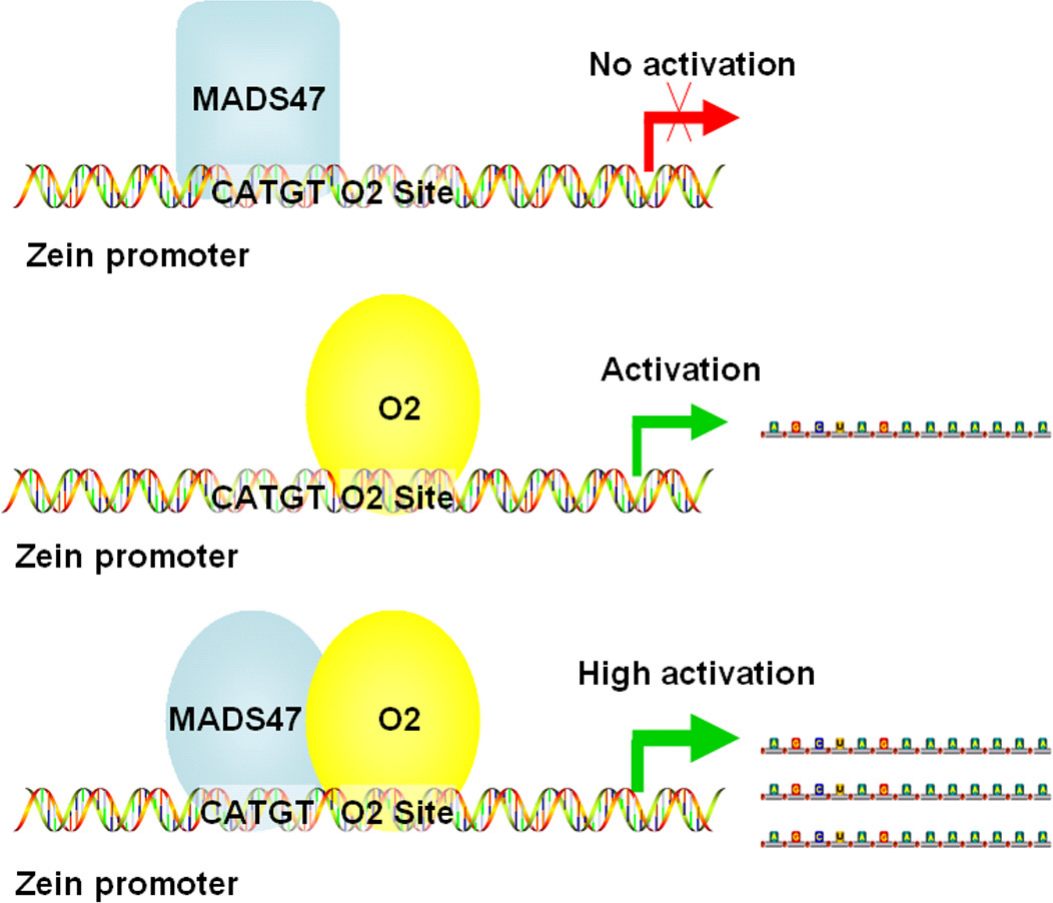
Illustrtion of how the O2/ZmMADS47 complex may regulate zein gene expression. The upper illustration shows ZmMADS47 alone (square) binds the CATGT motif but does not induce transactivation. The middle illustration shows O2 binding is able to transactivate its targets. The bottom illustration shows ZmMADS47 (oval) and O2 bind their respective motifs and the O2/ZmMADS47 complex enhances transactivation of gene targets.

To further explore whether ZmMADS47 can affect the transactivation of O2, a mutated ZmMADS47 with a seven amino acid deletion in the C-terminus was constructed (Fig 7C). The mutated ZmMADS47 (ZmMADS47 (Mu)) retained its ability to interact with O2 (S9 Fig); however, it’s C-terminal domain completely lost its transactivation ability (S10 Fig). When mutated ZmMADS47 and O2 were co-expressed in onion epidermal cells with the z1A promoter, no significant change was observed in the transactivation of the reporter gene compared to the expression level induced by O2 alone (Fig 7C). Therefore, the absence of AD function of ZmMADS47 was compensated by interaction with O2 and this interaction does not change the transactivation property of O2. Fig 8 summarizes the transcriptional regulation of the O2/ZmMADS47 protein complex.

## Discussion

### ZmMADS47 is a transcription factor for α and 50-kD γ-zein genes

Zeins are classified into α-, β-, γ- and δ-types, some of which are encoded by multigene families [2]. Previous work described several TFs, such as O2, that modulate zein gene expression [5]. The 22-kD α-zein genes are the best characterized targets of *O2* [29], but O2 also activates expression of 14-kD β-zein and 19-kD α-zein genes [31]. We have ablated the downstream targets of O2 by Chromatin Immunoprecipitation Sequencing (ChIP-Seq) technology and identified a vast number of zein genes as O2 targets [10]. However, expression of these genes responded differently to loss of O2 function in the *o2* mutant. Of note, expression of z1C (22-kD) α zeins and the 14-kD β-zein were severely reduced, while the 19-kD zeins were only partially reduced, and other zeins were barely affected [10]. This suggested regulation of zein genes is complex, and other TFs must exist. Additional TFs, including PBF and OHP1/OHP2, were identified as functional for 27-kD γ-zein gene regulation [15]. These studies also proved that TFs of zeins often interact with each other. PBF was shown to interact with O2 and OHP1/OHP2, and O2 can also interact with OHP1/OHP2 [14,15]. In this study, we describe another TF, i.e. ZmMADS47, which initially was identified as an O2-interacting protein (Fig 1). ZmMADS47 specifically binds a CATGT motif in the promoter of α zein and 50-kD γ-zein genes, and modulates their expression together with O2 (Fig 5).

MADS-box proteins comprise a large transcription factor family in plants. It contains two subfamilies, and ZmMADS47 belongs to the StMADS11-like subgroup [18]. Functional analysis showed MIKC-type MADS-box transcription factors are essential for regulation of various aspects of plant, especially sporophyte development and flower development [19,25]. There are also examples of MADS-box TFs regulating nutritional storage activities, but in an indirect way. For example, MADS-box transcription factor *OsMADS29* in rice, which also belongs to MIKC-type MADS-domain TFs, can indirectly affect starch granule biosynthesis via regulation of the cytokinin biosynthesis pathway [32]. Our results indicated that MIKC-type MADS-box transcription factor ZmMADS47 can affect nutritional reservoir activity by directly regulating storage protein genes in maize endosperm (Figs 3, 5 and 6).

In Zm*MADS47 RNAi* kernels, with down-regulation of α-zeins and 50-kD γ-zein, we observed many PBs are small and irregular in shape (Fig 4). Zein PBs have a peripheral layer rich in β- and γ-zein, while the α-zeins fill the center [33]. In earlier RNAi studies, knockdown of 22-kD α-zein led to irregular-shaped PBs, and knockdown of 27-kD γ-zein led to smaller and fewer PBs. 22-kD α-zein plays a critical function in PB expansion, with another observation indicating an important role for 27-kD γ-zein in PB initiation [28,34]. Other γ-zein family members function more in PB expansion rather than in PB initiation [35]. There was little research on the effect of 50-kD γ-zein knockdown alone on the shape of PBs to date. Zm*MADS47 RNAi* down-regulated 22-kD and 19-kD α-zeins, and 50-kD γ-zein. We did not observe a change in PB numbers between RNAi and counterpart WT endosperm cells, suggested that the PB initiation was not affected. However, only about 40% of PBs appeared expanded into normal size, whereas about 60% of PBs were poorly expanded and with irregular shapes. This could mainly due to the decrease of α-zeins, which impaired PB expansion. It only affected PB size and shape but did not render an obvious opaque kernel arising from a small reduction in zein content (16.8%, Fig 5) in Zm*MADS47 RNAi*. The phenotype of PBs in double mutant kernels was not enhanced compared to PBs in *o2* as we observed in Fig 4C and 4D, suggesting that the biological function of ZmMADS47 relies on the presence of O2 protein.

To date, four functional TFs for zein gene transcriptional regulation had been identified, i.e. O2, ZmMADS47, PBF and OHP1/OHP2. However, they cannot account for the transcriptional regulation of all zein genes, suggesting there are more unknown TFs to be discovered.

### ZmMADS47 was recruited to modulate zein expression during evolution

MIKC-type MADS-box transcription factors can be subdivided into several groups [18]. Among them, StMADS11-like MADS-box transcription factors have highly diverged functions in plant development. Two closely related *Arabidop*sis StMADS11-like MADS-box genes, *AGL24* and *SVP*, are examples of genes controlling floral transition. While AGL24 acts as a promoter of this process [36], SVP is a repressor of flowering [37]. *JOINTLESS*, from tomato, modulates flower and fruit development [38]. OsMADS47, the homologue of ZmMADS47 in rice, was reported to modulate floral reversions and brassinosteroid responses [25]. ZmMADS19 (TU1) shares high sequence similarity with ZmMADS47 (S3B Fig) and influences inflorescence architecture by promoting indeterminate cell fate [39]. StMADS11-like MADS-box TFs function in regulating timing of different stages of vegetative development or the transition to reproductive development. According to expression profile analysis, ZmMADS47 is not an endosperm-specific TF (S3C and S3D Fig). Using the kernel-specific transgenic Zm*MADS47 RNAi* experiment, we uncovered ZmMADS47’s function in zein gene transcriptional regulation during maize endosperm development (Figs 3 and 6). Nonetheless, the exact function of ZmMADS47 in other tissues is still unclear, and additional investigation will be needed to unveil its other biological functions.

Phylogenetic analysis indicated that zein genes have a relatively recent evolutionary origin. The oldest α zein gene family, z1D, arose in around 21–26 million years ago (MYA) [40]. The MADS-box gene family appears to be very ancient. Type II MADS-box TFs first emerged in charophytes (*Selaginalla moellendorfil*) around 700 MYA, and StMADS11-like MADS-box TFs emerged in at least 300 MYA, which makes them much older than the α zein gene family [18]. Considering the relative new origin of zein gene families, it is not surprising that older transcription factors, such as ZmMADS47, would be adopted into their transcriptional regulatory network.

MADS-type proteins prefer to bind the CArG box according to earlier studies [41]. But SEPALLATA3, a MADS-box DNA-binding protein in *Arabidopsis* flowers, can recognize the non-CArG-type G-box (bHLH/bZIP) and CCNGGG (TCP) motifs *in vivo* [42]. We found that ZmMADS47 also binds non-CArG-type motif with core sequence CATGT in α zein and 50-kD γ zein promoters (Fig 5), suggesting that ZmMADS47 is recruited to zein promoters, next to the O2 protein (Fig 5). TFs usually bind many more DNA sites in genomes than predicted, most of which are non-functional [43]. However, in the case of ZmMADS47, it appears a new biological function was acquired after binding a zein promoter and interacting with O2. This interaction could increase zein content in mature seeds due to enhanced transcriptional activity. Therefore, this novel protein-DNA and protein-protein interaction could have been functionally selected during evolution; because higher storage protein content would be beneficial for seed germination and seedling survival.

### ZmMADS47 is functionally modulated by a conformation modulated mechanism

As transcriptional regulators, MIKC-type MADS-box TFs often display entirely different roles in gene regulation. In some cases, MIKC-type MADS proteins are repressors, but in others, these proteins act as activators [44]. MIKC-type MADS-box TFs can also bind other MIKC-type proteins. In maize, BDE can form a protein complex with ZAG1, also a MIKC-type MADS-box TF, and can modulate floral organ number and development [45]. OsMADS47 interacts with OsMADS22 and other MIKC-type MADS-box proteins that interfere with the formation of complexes required for normal flower development [24]. MIKC-type TFs can also interact with other TF family proteins. For example, OsMADS57 can interact with OsTB1, a TCP transcription factor controling tillering [41]. But MIKC-type TFs have not been reported to interact with bZIP TFs as instead shown in this study (Fig 1).

There are multiple reports of conformational changes in TFs resulting in novel functions. Of note, *Aarabidopsis* MYC3 changes its conformation after binding a conserved Jas motif in the JAZ9 repressor and modulates repression of the jasmonate signaling pathway [46]. Other work demonstrated the C-terminal region of MIKC-type MADS-box proteins, which is composed of hydrophobic amino acids, may play a critical function in transactivation [47]. In this study, we found that full-length ZmMADS47 could not activate reporter gene expression in yeast and plant cells, but the C-terminus of ZmMADS47 has strong transactivation capability (Figs 2C and 6). This result suggests the transactivation domain of ZmMADS47 is inhibited in its full-length native form, implying a unique regulatory mechanism to acquire this hidden transactivation ability. Our results clearly showed that after interacting with O2, the transcriptional activity of ZmMADS47 was released (Fig 7). This protein-protein interaction most likely caused a conformational change of ZmMADS47, and that opened its C-terminal transactivation domain (Fig 8).

O2 is specifically expressed in maize endosperm. However, ZmMADS47 is expressed in many other tissues, such as root, stalk and husk. Our data suggest that to act as a functional TF, ZmMADS47 must interact with other partner(s) to achieve a similar conformational change. Because ZmMADS47 interacts with the bZIP domain (O2-2) of O2, it is possible that other bZIP TFs display similar protein-protein interactions with the other MADS-box proteins. It is also possible that ZmMADS47 may interact with other proteins to achieve similar conformational change for releasing the transactivation activity.

## Materials and Methods

### Plant materials

The *o2* mutant stock (701D) and HiII maize transformation parental lines PA and PB were initially obtained from the Maize Genetics Cooperation Stock Center, and maintained in the lab. The Zm*MADS47 RNAi* transgenic lines were generated in the lab. Maize inbred lines W22 and B73 were maintained in the lab. Maize plants were cultivated in the experimental field, green house or growth chamber at the campus of Shanghai University, Shanghai.

### Yeast two hybridization assay

The full-length *ZmMADS47* open reading frame (ORF) was amplified with gene-specific primers and cloned into *Nco*I and *Sal*I sites of pGAD-T7 vector (Clontech). pGBK-T7-*O2(bZIP)* was constructed according to published before [21]. The pGAD-T7-*ZmMADS47* and pGBK-T7*-O2(bZIP)* as prey and bait vectors were both transformed into AH109 and cultured until OD_600_ reached 0.5. AD+ZmMADS47 and O2(bZIP)+AD were used as negative controls. Different yeast strains were all grown on DDO (SD/-Leu/-Trp) plate and QDO (SD/-Leu/-Trp/-Ade/-His) plate containing 10 mM 3-AT (3-amino-1, 2, 4-triazole) (Sigma Aldrich).

### Pull-down assay

For generating His-tagged O2 protein, O2 ORF was amplified and cloned into *Not*I/*Nco*I sites of pET-32a (Novagen). For generating GST-tagged ZmMADS47 and GST-tagged ZmMADS47(Mu) protein, *ZmMADS47* ORF and truncated *ZmMADS47* (1–678 bp) were amplified and cloned into *BamH*I/*Xho*I sites of pGEX-4T-1 (Amersham Biosciences). Recombinant proteins were induced following the manufacturer’s manual. For GST Pull-down assay, approximately 20 μg *E*. *coli* lysates containing GST or GST-ZmMADS47 fused protein were mixed with 25 μl Immobilized Glutathione Agarose (Thermo Scientific), for more than 30 min at 4°C. Then, the beads were washed 5 times, with 400 μl washing buffer. The washed beads were then incubated with His or His-O2 fused protein lysates for more than 2 hours at 4°C. The protein complex-Immobilized Glutathione Agarose conjugates were eluted in 50 μl Glutathione Elution Buffer containing 100 mM Glutathione (Thermo Scientific). The bound proteins were analyzed by 10% PAGE and western blot with His or GST antibodies (Abcam).

### Polyclonal antibody preparation

To generate antibody specific to ZmMADS47, cDNA fragment containing the first 400 bp from 5’ ORF was cloned into the pGEX-4T-1 GST-tagged vector by *BamH*I/*Xho*I sites. Recombinant protein were expressed in bacterial strain BL21 (DE3) (Novagen) by adding 500 μM IPTG (Dingguo) to a final concentration of 0.4 mM under 25°C, and purified using the ÄKTA purifier system (GE Healthcare). The antibody was prepared by Shanghai ImmunoGen Biological Technology in rabbit according to standard protocol. To generate antibodies specific to O2, O2 ORF was amplified and cloned into *Not*I/*Nco*I sites of pET-32a (Novagen). His-O2 fused protein was induced in *E*. *coli* strain Rosetta (DE3), and purified by the ÄKTA purifier system (Amersham Biosciences). The antibodies were prepared by Shanghai ImmunoGen Biological Technology according to standard protocol. The 19-kD, 22-kD α-zein, 16-kD, 27-kD, 50-kD γ-zein, 14-kD β-zein, 10-kD δ-zein specific antibodies were produced according to the protocol published before [4].

### Co-immunoprecipitation

Total kernel protein was extracted by grounding in liquid nitrogen and suspending in extraction buffer (50 mM Tris-Cl, 2.5 mM EDTA, 150 mM NaCl, 0.2% NP-40, 20% glycerol, 1 mM PMSF and 1% plant cocktail [Sigma Aldrich]) on ice for more than 20 minutes. The lysate was then centrifuged at 12,000 rpm for 5 minutes, discarded the pellet, and centrifuged one more time. For co-immunoprecipitation, the protein lysate was incubated with 5 μl O2 antibody from guinea pig for 2 hours at least. Then the protein-antibody complex was incubated with 100μl ProteinA Sepharose CL-4B (GE Healthcare) for at least 30 minutes. After washing with extraction buffer for 4 times, western blot was carried out with O2 (1:1000 dilution) and ZmMADS47 (1:1000 dilution) antibodies.

### Gel filtration chromatography

Total kernel protein was extracted as described in co-immunoprecipitation method. A Superdex 200 10/300 GL Column (GE Healthcare) was firstly equilibrated in protein extraction buffer with an ÄKTA purifier system (GE Healthcare) until the UV baseline was smooth. 500 μl of maize kernel lysate was injected into the system and the flow speed adjusted to 0.5 ml per minute. After the first volume (6 ml) was flowed, consecutive fractions of 500 μl each were started to be collected. The collected fractions were then concentrated by acetone and analyzed by SDS-PAGE and western-blot.

### Phylogenetic analysis

For phylogenetic analysis, 10 most related protein sequences to ZmMADS47 in maize (*Zea mays*) or rice (*Oryza sativa*) were selected by employing NCBI BLASTp search using nr (non-redundant protein sequences) database. Their corresponding GenBank/EMBL accession numbers as following: *ACG34827*.*1*, *NP_001105148*.*1*, *NP_001105154*.*1*, *CAD23411*.*1*, *ACG36350*.*1*, *NP_001104926*.*1*, *NP_001105332*.*1*, *NP_001105153*.*1*, *NP_001288437*.*1*, *NP_001105150*.*1*, *AAQ23142*.*1*, *BAD15933*.*1*, *BAD29571*.*1*, *AAK26241*.*1*, *AAF04972*.*1*, *AAK26241*.*1*, *AAS59823*.*1*, *P0C5B2*.*1*, *AAS59832*.*1*, *AAS59829*.*1*. All these 20 sequences were aligned with MUSCLE method in MEGA6.06 software. The evolutionary distances were computed using the Poisson correction analysis. The bootstrap method was used 1000 replicates for phylogeny test [48].

### RNA extraction and quantitative PCR

Totally RNA was extracted from developing kernels and other tissues using RNA extraction kit (TIANGEN) and synthesized cDNA by reverse transcriptional kit (Roche). Primer pairs for quantitative PCR were all designed using Quantiprime software (http://www.quantprime.de). Ubiquitin gene (GenBank Accession Number: BT018032) was used as internal control. For quantitative PCR, the reaction mixture was composed by cDNA first-strand template, primer mix and SYBR Green Mix (Roche) to a final volume of 20 μl. The reactions were performed using a Mastercycler ep realplex 2 (Eppendorf). All results were repeated 3 times. The data was analyzed by ∆∆Ct method as previously described [21].

### Sub-cellular localization

Full length (1–702 bp) or different fragments (MADS box: 1–234 bp; K box: 235–528 bp; C: 529–702 bp) of *ZmMADS47* ORF were cloned into transit expression vector pSAT6-EYFP-N1 at *Nco*I/*Sal*I sites, and the enhanced yellow fluorescent protein (EYFP) was substituted by enhanced cyan fluorescent protein (ECFP) using *Xba*I and *BamH*I sites [49]. Living onion (*Allium cepa*) epidermal cells were peeled and cultured on MS media in dark for more than 8 hours. One μg plasmid of each construct was used to coat 0.3 mg of 1.0-μm-diameter tungsten particles, and bombarded into the cells with the Biolistic PDS-1000/He System (Bio-Rad). The bombarded samples were cultured for more than 12 hours in dark. The samples were then observed using laser confocal microscope (LSM710, Zeiss).

### Transactivation assay in yeast

For transactivation assay in yeast, full length (1–702 bp) or different fragments (MADS box: 1–234 bp; K box: 235 bp-528 bp; C: 529–702 bp; C(Mu):529–678 bp) of ZmMADS47 ORF were cloned into yeast expression binary vector pGBK-T7 at *Nco*I and *Sal*I sites, and then co-transformed into yeast strain EGY48 with pG221. The pG221 vector contains a minimal promoter which could be bound by DNA binding motif from pGBK-T7 vector. The pGBK-T7-*O2* containing full length O2 ORF was used as positive control while the empty vector pGBK-T7 was used as negative control. The yeast cells were cultured in DDO (SD/-Leu/-Ura) liquid medium until OD600 reached 0.5–0.8. Then the cells were collected by centrifugation (12,000rpm), and resuspended in 0.1 mL Z buffer (60 mM Na_2_HPO_4_·7H_2_O, 40 mM NaH_2_PO_4_·H_2_O, 10 mM KCl, 1 mM MgSO_4_·7H_2_O, pH7.0), and busted in 37°C and liquid nitrogen cycle. The tubes were consecutively added 0.7mL Z buffer containing mercaptoethanol and 0.16 mL o-Nitrophenyl-β-D-Galactopyranoside (ONPG) which dissolved in Z buffer at a final concentration of 4 mg/mL. The mixuture was incubated in 30°C until the products synthesized. Finally, 0.4 ml 1M Na_2_CO_3_ was added to stop the reaction and the products were analyzed at OD_420_ using spectrophotometer (BioPhotometer Plus, Eppendorf).

### Zm*MADS47 RNAi* knockdown lines construction

A cDNA fragment contianing the 200–500 bp from 5’ ORF of *ZmMADS47* was selected as interference fragment according to http://rnaidesigner.thermofisher.com/rnaiexpress/. A *Nco*I/*Swa*I fragment containing reverse orientated fragment and a *BamH*I/*Xba*I fragment containing forward orientated fragment were cloned into pFGC-5941 vector at corresponding sites [50]. The cauliflower mosaic virus 35S promoter in pFGC-5941 was replaced by a 16-kD γ-zein promoter (2031 bp upstream to ATG) to provide maize endosperm specific expression. The construct was transformed into *Agrobacterium tumefaciens* (GV3101). Agrobacterium mediated maize transformation was carried out according to published protocol [51]. Five independent transgenic lines were generated. Southern blot analysis using BAR gene probe was performed to estimate transgenic copy numbers using T0 generation genomic DNAs according to standard procedures. The transgenic lines were all backcrossed into W22 for three generations.

For further analysis, 15 DAP kernels from selfed heterozygous RNAi line 3 were genotyped by PCR assay using RNAi construct-specific primers. Non-transgenic kernels were pooled as the WT sample, while transgenic kernels were pooled as the RNAi sample. Total RNA was extracted by using RNAprep Pure Plant Kit (TIANGEN) for real-time PCR or RNA-Seq.

### Southern-blot analysis

About 10 μg genomic DNA were digested by 40 units of *Eco*RI in 37°C for 4 hours. DNA fragments were seperated by electrophoresis in 1% (w/v) agarose gel over night at 4°C and blotted to nylone membrane (GE Healthcare) accoridng to standard protocal. DNA probe was labeled by DIG High Prime DNA labeling kit (Roche), and hybridized with DNA blot by standard procedure. Signal was detected using DIG Detection Starter kit (Roche) according to manufacture’s protocol.

### Measurement of total proteins, zeins and non-zeins

Mature kernels of WT or RNAi line 3 and line 6 were collected from well-filled ears. After genotype identification, total proteins, zeins and non-zeins were all extracted from 50 mg of dried endosperm flour according to published before [52]. Quantification of the total proteins, zeins and non-zeins were performed as described before [53]. SDS-PAGE was performed on 12% polyacrylamide gels and visualized by staining with Coomassie brilliant blue (Dingguo).

### Immunoblot analysis

Proteins were separated by SDS-PAGE, and transferred to polyvinylidene difluoride membranes (0.45 mm; Amersham Biosciences). The membranes were incubated with primary and secondary antibodies. Amersham ECL Prime Western Blot Detection Reagent (GE Healthcare) was used to visualize the signal. The 22-kD α-zein antibody was used at 1:100, 16-kD-, 27-kD-, 50-kD γ-zein, 14-kD β-zein, 10-kD δ-zein antibodies were used at 1:500. The ZmMADS47, O2 and 19-kD α-zein antibody was used at 1:1000. The α-tubulin (Sigma-Aldrich), α-GST, α-His antibody and secondary antibodies (Abcam) was used at 1:5000.

### RNA-seq analysis

Total RNA (10 μg) was extracted from immature kernels of Zm*MADS47 RNAi* lines and non-transgenic control lines at 15 DAP. The RNA-seq libraries were prepared according to Illumina standard instruction (TruSeq Standard RNA LT Guide) followed by quality check with Agilent 2100 bioanalyzer, and sequenced on an Illumina HiSeq 2000 according to manufacturer’s instructions (HiSequation 2000 User Guide). Effective reads were aligned to Ensembl plants release-15 zea_mays genome build Zea_mays.AGPv2.15 using Tophat software [54]. The unique reads were normalized as fragments per kilobase of exon per million fragments mapped (FPKM) using Cufflink (Version:2.1.1) [55]. Differential expression genes were defined as those with changes >1.4 or <0.71 and P<0.05.

### Transmission electron microscopy

Immature kernels at 18-DAP were prepared according to described protocol [56], with some modifications: the kernels were soaked in paraformaldehyde and post fixed in osmium tetraoxide. Fixed samples were dehydrated in an ethanol gradient up to 100% and then transferred to a propylene oxide solution and slowly embedded in acrylic resin (London Resin Company). Thin sections (70 nm) were made using a diamond knife microtome (Reichert Ultracut E). Sections were placed on 100-mesh copper grids and stained for 30 min with uranyl acetate and for 15 min with lead citrate. Sections were observed with a transmission electron microscope (Hitachi H7600).

### Number and size measurement of protein body

The TEM images of the sub-cellular morphology of the fourth and fifth starchy endosperm cell layers (1,500× and 3,000×) were loaded by Image Pro Plus 6.0 (Media Cybernetics). Number of protein bodies in the images (1,500×) was counted. Area of protein bodies in the images (3,000×) was measured. After export from Image Pro Plus, the data was analyzed using GraphPad Prism (Version 6.0c).

### Electrophoretic mobility shift assay (EMSA)

The His-ZmMADS47 fusion protein in pET-32a vector as described in pull-down assay, was expressed and purified with TALON Resin (GE Healthcare) and used for EMSA. Oligonucleotide probes (S2 Table) were synthesized and labeled according to the standard protocol by Shanghai Invitrogen Technology. Standard reaction mixture for EMSA contained 20 ng of purified His-ZmMADS47 fusion protein, 5 ng of biotin-labeled annealed oligonucleotides, 2 μl of 10× binding buffer (100 mM Tris, 500 mM KCl, and 10 mM DTT, pH 7.5), 1 mL of 50% (v/v) glycerol, 1 mL of 100 mM MgCl_2_, 1 mL of 1 mg/mL poly(dI-dC), 1 mL of 1% (v/v) Nonidet P-40, and double-distilled water to a final volume of 20 mL. The reactions were incubated at 25°C for 20 min, and electrophoresed on 8% (w/v) polyacrylamide gels, and then transferred to N+ nylon membranes (Millipore). Biotin-labeled DNA was detected using the LightShift Chemiluminescent EMSA kit (Thermo Scientific). Signals were visualized by X-ray film exposure.

### Transient assays for in vivo activation activity

The reporters were constructed based on pGreenII 0800-LUC vector [30] and the effectors were constructed based on pUC18-35S vector. To generate the pGreenII Pro:zein-LUC and pGreenII Pro:zein(Mu)-LUC reporters for the dual-luciferase assays, the -500 bp promoter fragments from TSS of different zein genes were amplified from W22 genomic DNA by PCR. To clone into pGreenII 0800-LUC vector, *Hind*III and *Not*I sites were used for 19-kD z1A, z1B, z1C, z1D and 14-kD zein promoters; *Xho*I and *Not*I sites were used for 27-kD zein promoter; while *Sal*I and *BamH*I sites were used for 10-kD and 50-kD zein promoters, respectively. The 50-kD γ-zein promoter with deleted O2 recognition site (Pro: zein(Mu) was cloned into pGreenII 0800-LUC with *Sal*I and *BamH*I sites.

To construct the CaMV 35S promoter-driven O2 and ZmMADS47 effector (pUC-35S-O2, pUC-35S-ZmMADS47), the ORFs of *O2* and *ZmMADS47* were cloned into the *Sma*I and *EcoR*I sites of pUC18-35S. To construct the CaMV 35S promoter-driven O2(Mu) effector pUC-35S-O2(Mu), the truncated *O2* (229–1374 bp) was amplified by PCR and cloned into pUC-35S using *Sma*I and *EcoR*I sites. To construct ZmMADS47(Mu) effector pUC-35S-ZmMADS47(Mu), the partial ORF of *ZmMADS47* without last 24 bp was cloned into pUC-35S using *Sma*I and *EcoR*I sites.

Transient dual-luciferase assays in onion (Allium cepa) epidermal cells were performed and measured using dual-luciferase assay reagents (Promega). Briefly, 1 μg of DNA was used to coat 0.3 mg of 1.0-μm-diameter tungsten particles. The onion epidermal cells were bombarded using the PDS-1000 system (Bio-Rad) at 1,100 p.s.i. helium pressure twice and then the bombarded samples were incubated for at least 8 h in the dark at 25°C. Sample discs (1 to 2 cm in diameter) were ground in liquid nitrogen and homogenized in 100 mL passive lysis buffer from the dual-luciferase assay reagents (Promega). The crude extract (20 mL) was mixed with 100 mL of luciferase assay buffer and the firefly luciferase (LUC) activity measured using the Tecan M200 system. One hundred microliters of Stop and Glow Buffer was then added to the reaction and Renilla luciferase (REN) activity was measured. Three independent measures were carried out for each analysis.

### Accession numbers

The genes mentioned in this article can be found in GenBank data library from national Center of Biotechnology information (NCBI). The accession number of ZmMADS47 is ACG34827.1. The accession number of Opaque2 is NP_001105421.2. The accession number of ZmUBQ is BT018032. Gene expression data that performed by using next-generation RNA sequencing technology can be acquired from GEO with accession numbers GSE70609.

## Supporting Information

S1 Fig BLASTP alignment of ZmMADS47 and OsMADS47. BLASTP alignment of ZmMADS47 and OsMADS47. The upper lines represent ZmMADS47 protein sequence and the lower lines represent OsMADS47 protein sequence. The shaded residues mean the same sequences of two proteins. The color residues mean the diversity of two proteins.
(PDF)

S2 Fig Western blot showing the specificity of O2-specific and ZmMADS47-specific antibodies. The extracts of 18-DAP immature kernels were blotted with O2-specific and ZmMADS47-specific antibodies, respectively. NC: Negative control with no extract. Extract: The extracts of 18-DAP immature kernels. IB: immunoblot.
(PDF)

S3 Fig Bioinformatics analysis and expression pattern of ZmMADS47. **A.** Structure of ZmMADS47. The *ZmMADS47* gene contains 8 exons and the protein has 233 amino acids with three motifs: MADS-box (N), K-box (K) and C terminal (C). **B.** Phylogenetic tree of ZmMADS47. **C.** Expression patterns of *ZmMADS47* RNA in different tissues and differential stages of kernel development. * denotes the embryo and endosperm from 15DAP kernels. Error bars represent SD (n = 3). **D.** ZmMADS47 protein levels in different tissues and different kernel development stages. α-tubulin antibody was used as an internal control. **E.** Expression levels of *O2* RNA in different tissues and stages of kernel development. * denotes the embryo and endosperm from 15DAP kernels. Error bars represent SD (n = 3). **F.** O2 protein level in different tissues and different kernel development stages. α-tubulin antibody was used as an internal control.
(PDF)

S4 Fig RNAi knockdown of *ZmMADS47* expression. **A.** Schematic representation of Zm*MADS47 RNAi* transgene construct. pFGC-5941 RNAi vector was used for construction. **B**. Southern hybridization analysis of transformants in five Zm*MADS47 RNAi* transgenic lines. Five independent lines (line 3, line 6, line 7, line 8, line A) showed specific transgene insertions. About 10 μg genomic DNA were digested by 40 units of *Eco*RI in 37°C for 4 hours before DNA electrophoresis. NC: Negative control. **C.** Analysis of *ZmMADS47* RNA expression in different RNAi lines by qRT-PCR. Gray bars represent the expression of *ZmMADS47* in wild type lines. Black bars represent the expression of *ZmMADS47* in RNAi lines (line3, line6, ling7, line8, lineA). Error bars represent SD (n = 3) (*P < 0.05, **P < 0.01, Student’s t test). **D.** Western blot showing the ZmMADS47 protein levels in RNAi lines 3 and 6. α-tubulin antibody was used as the internal control.
(PDF)

S5 Fig Transmission electron microscope of the wild type and Zm*MADS47RNAi* kernels. **A,B.** Protein bodies were observed by transmission electron microscope in wild type (A) and Zm*MADS47 RNAi* (B) 18-DAP kernels. Each genotype is labeled above the corresponding TEM images. PB: protein body; CW: cell wall; SG: starch granule. Bars represent 5 μm.
(PDF)

S6 Fig The His tag in His-ZmMADS47 does not affect the DNA-binding pattern of the recombinant protein. Purified His-tag was used as negative control.
(PDF)

S7 Fig Schematic representation of ZmMADS47 and O2 DNA binding sites in z1A α zein promoter and 50-kD γ zein promoter. The blue letters represent TATA box in z1A α-zein promoter and 50-kD γ-zein promoter.
(PDF)

S8 Fig Transactivation ratio of different zein genes. LUC/REN is the ratio of luciferase activity and reniformis activity. Error bars represent SD (n = 6) (*P < 0.05, **P < 0.01, ***P < 0.001, Student’s t test). y-axis represents the ratio of LUC/REN.
(PDF)

S9 Fig pull-down assay analysis of ZmMADS47(Mu) and O2. GST antibody and Opaque2 antibody were used to detect ZmMADS47(Mu)-GST fusion protein and Opaque2, respectively.
(PDF)

S10 Fig ZmMADS47 with truncated C terminal activation domain was used to test the transactivation ability in yeast EGY48 strain. Error bars represent SD (n = 3) (***P<0.001, Student’s t test).
(PDF)

S1 Table Gene ontology classifications of DEGs with functional annotation in Zm*MADS47 RNAi* transgenic line. (PDF)

S2 Table Probes used for EMSA. (PDF)

S3 Table Primers involved in this paper. (PDF)
