# Supplementary material for: ZmMADS47 Regulates Zein Gene Transcription through Interaction with Opaque2
Source: PLoS Genet. 2016 Apr 14;12(4):e1005991. doi: 10.1371/journal.pgen.1005991 (PMC4831773; doi:10.1371/journal.pgen.1005991)
Supplement: S1 Fig — BLASTP alignment of ZmMADS47 and OsMADS47. The upper lines represent ZmMADS47 protein sequence and the lower lines represent OsMADS47 protein sequence. The shaded residues mean the same sequences of two proteins. The color residues mean the diversity of two proteins. (PDF) [file pgen.1005991.s001.pdf]

S1 Fig

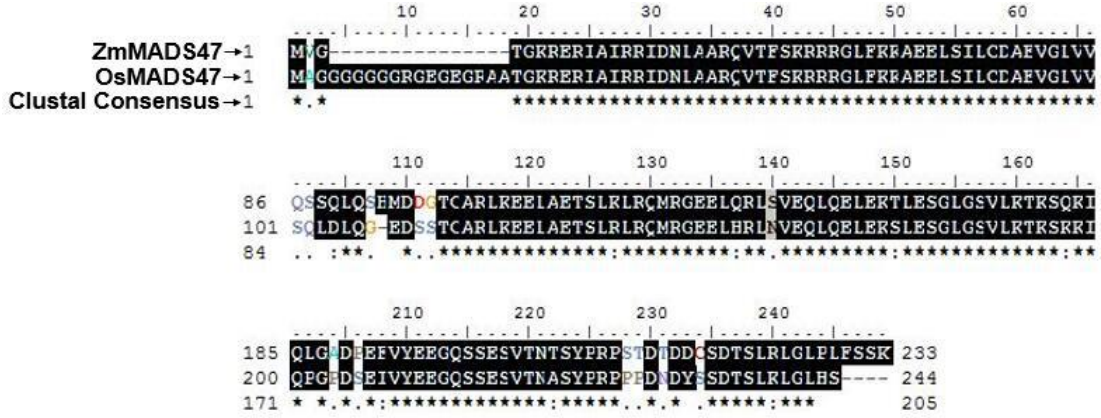
ZmMADS47→1
OsMADS47→1
Clustal Consensus→1
10 20 30 40 50 60
MVG----------TGRRERIAIRRIDNLAARÇVTFSKRRRGLFKKAEELSILCDAEVGLVV
MAGGGGGGGRGEGEGFAATGRRERIAIRRIDNLAARÇVTFSKRRRGLFKKAEELSILCDAEVGLVV
. **********
110 120 130 140 150 160
86 QSSQLQSEMDDGTCARLKEELAETSLRLRÇMRGEELQRLSVEQLQELERTLESGLGSVLRTKSQRI
101 SQLDLQG-EDSSTCARLKEELAETSLRLRÇMRGEELHRLNVEQLQELEKSLESGLGSVLRTKSKRI
84 .. :**. *..**********:*********:**.*********:**********:**
210 220 230 240
185 QLGSDPEFVYEEGQSSESVTNTSYPRPSTDTDDGSDTSLRLGLPLFSSK 233
200 QPGPDSEIVYEEGQSSESVTNASYPRPPPDNDYPSDTSLRLGLHS---- 244
171 * *.*.*:**********:*****..*.* .*****:*** 205
