# Supplementary figures and images for: ZmMADS47 Regulates Zein Gene Transcription through Interaction with Opaque2
Source: PLoS Genet. 2016 Apr 14;12(4):e1005991. doi: 10.1371/journal.pgen.1005991 (PMC4831773; doi:10.1371/journal.pgen.1005991)

**S2 Fig**

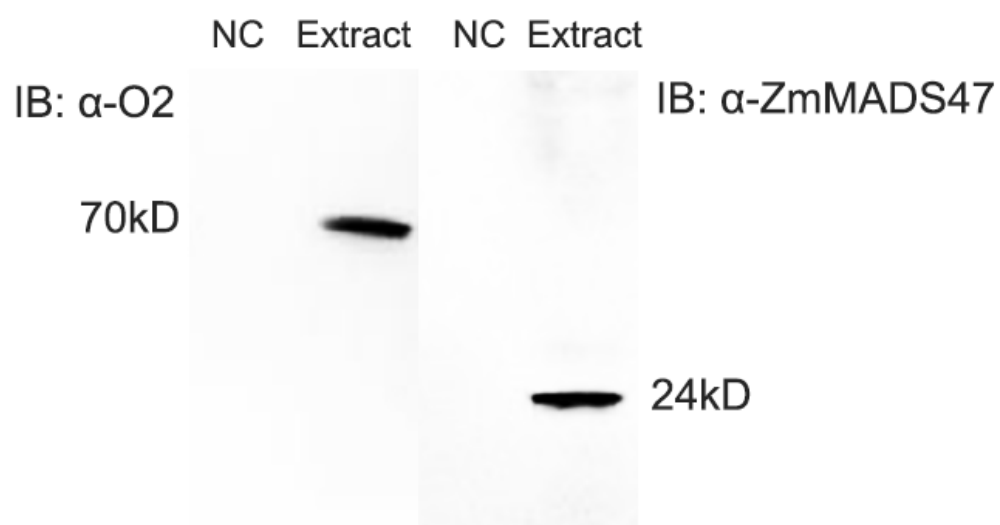

Supplement: S2 Fig — The extracts of 18-DAP immature kernels were blotted with O2-specific and ZmMADS47-specific antibodies, respectively. NC: Negative control with no extract. Extract: The extracts of 18-DAP immature kernels. IB: immunoblot. (PDF) [file pgen.1005991.s002.pdf]

**S3 Fig**

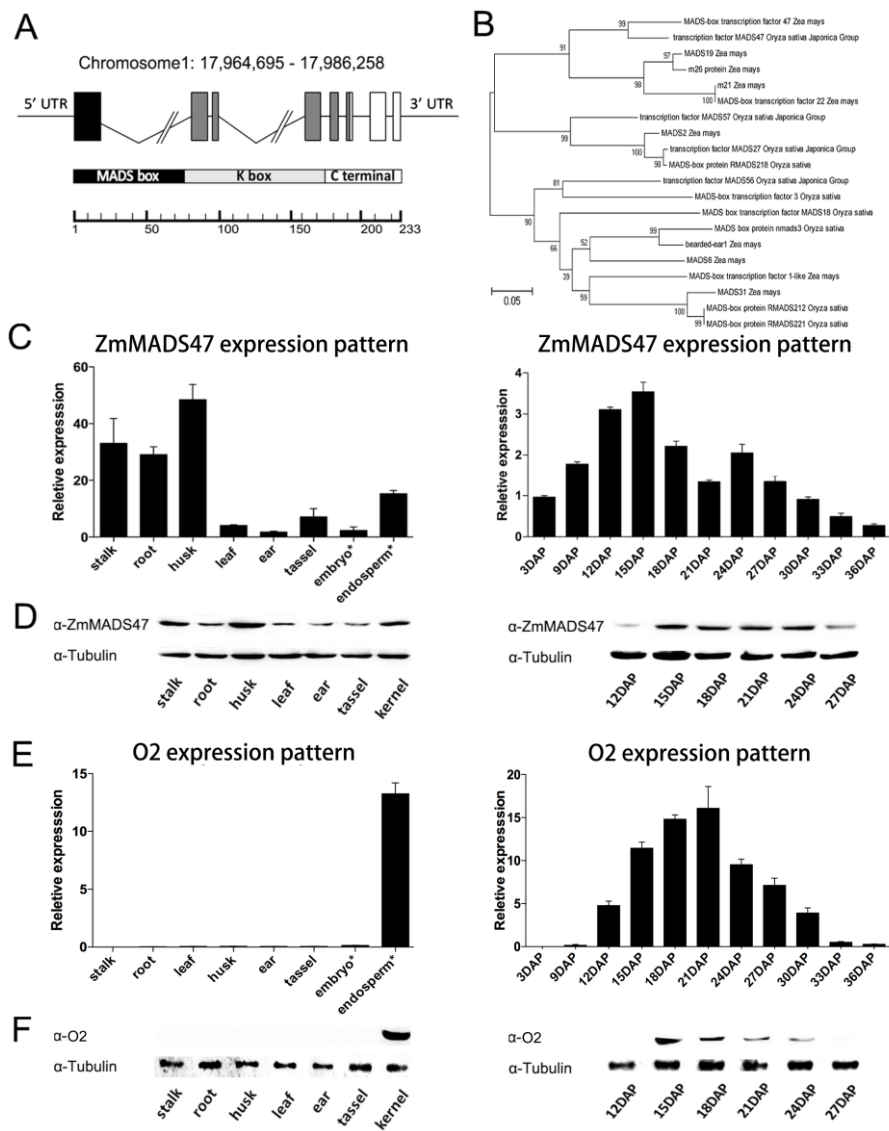

Supplement: S3 Fig — A. Structure of ZmMADS47. The ZmMADS47 gene contains 8 exons and the protein has 233 amino acids with three motifs: MADS-box (N), K-box (K) and C terminal (C). B. Phylogenetic tree of ZmMADS47. C. Expression patterns of ZmMADS47 RNA in different tissues and differential stages of kernel development. * denotes the embryo and endosperm from 15DAP kernels. Error bars represent SD (n = 3). D. ZmMADS47 protein levels in different tissues and different kernel development stages. α-tubulin antibody was used as an internal control. E. Expression levels of O2 RNA in different tissues and stages of kernel development. * denotes the embryo and endosperm from 15DAP kernels. Error bars represent SD (n = 3). F. O2 protein level in different tissues and different kernel development stages. α-tubulin antibody was used as an internal control. (PDF) [file pgen.1005991.s003.pdf]

A

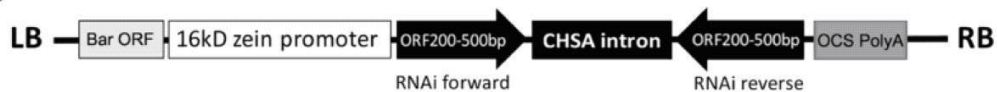

B

NC line 3 line 6 line 7 line 8 line A

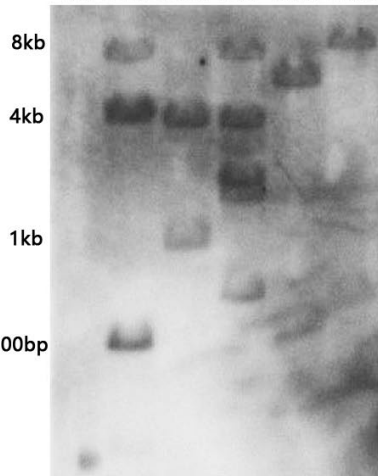

C

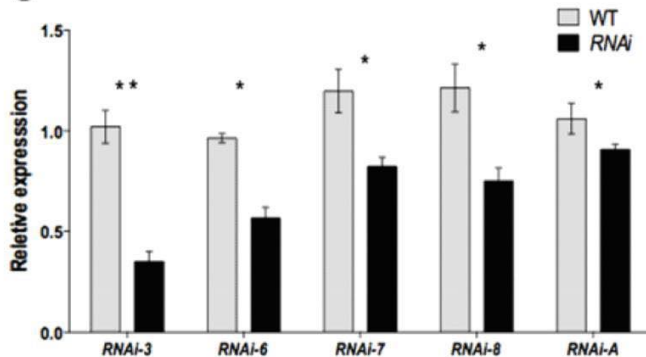

D

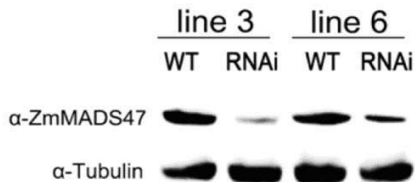

Supplement: S4 Fig — A. Schematic representation of ZmMADS47 RNAi transgene construct. pFGC-5941 RNAi vector was used for construction. B. Southern hybridization analysis of transformants in five ZmMADS47 RNAi transgenic lines. Five independent lines (line 3, line 6, line 7, line 8, line A) showed specific transgene insertions. About 10 μg genomic DNA were digested by 40 units of EcoRI in 37°C for 4 hours before DNA electrophoresis. NC: Negative control. C. Analysis of ZmMADS47 RNA expression in different RNAi lines by qRT-PCR. Gray bars represent the expression of ZmMADS47 in wild type lines. Black bars represent the expression of ZmMADS47 in RNAi lines (line3, line6, ling7, line8, lineA). Error bars represent SD (n = 3) (*P < 0.05, **P < 0.01, Student’s t test). D. Western blot showing the ZmMADS47 protein levels in RNAi lines 3 and 6. α-tubulin antibody was used as the internal control. (PDF) [file pgen.1005991.s004.pdf]

**S5 Fig**

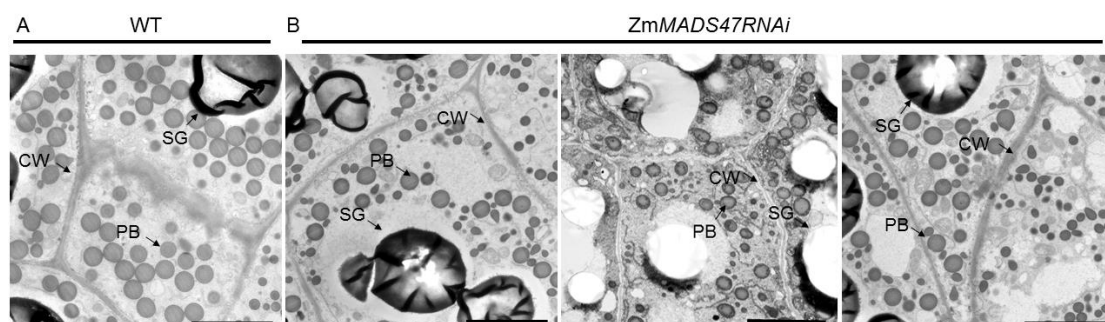

Supplement: S5 Fig — A,B. Protein bodies were observed by transmission electron microscope in wild type (A) and ZmMADS47 RNAi (B) 18-DAP kernels. Each genotype is labeled above the corresponding TEM images. PB: protein body; CW: cell wall; SG: starch granule. Bars represent 5 μm. (PDF) [file pgen.1005991.s005.pdf]

**S6 Fig**

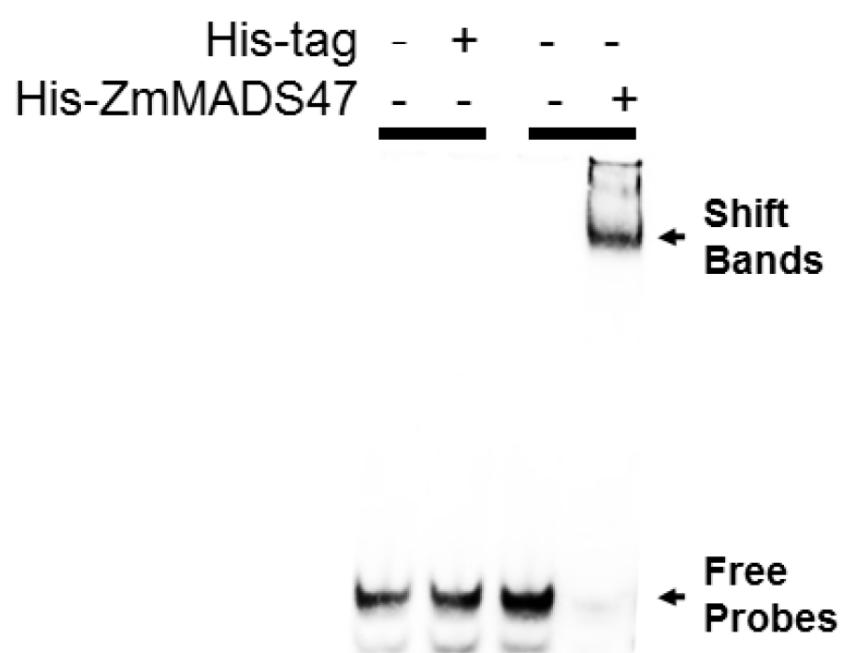

Supplement: S6 Fig — Purified His-tag was used as negative control. (PDF) [file pgen.1005991.s006.pdf]

S8 Fig

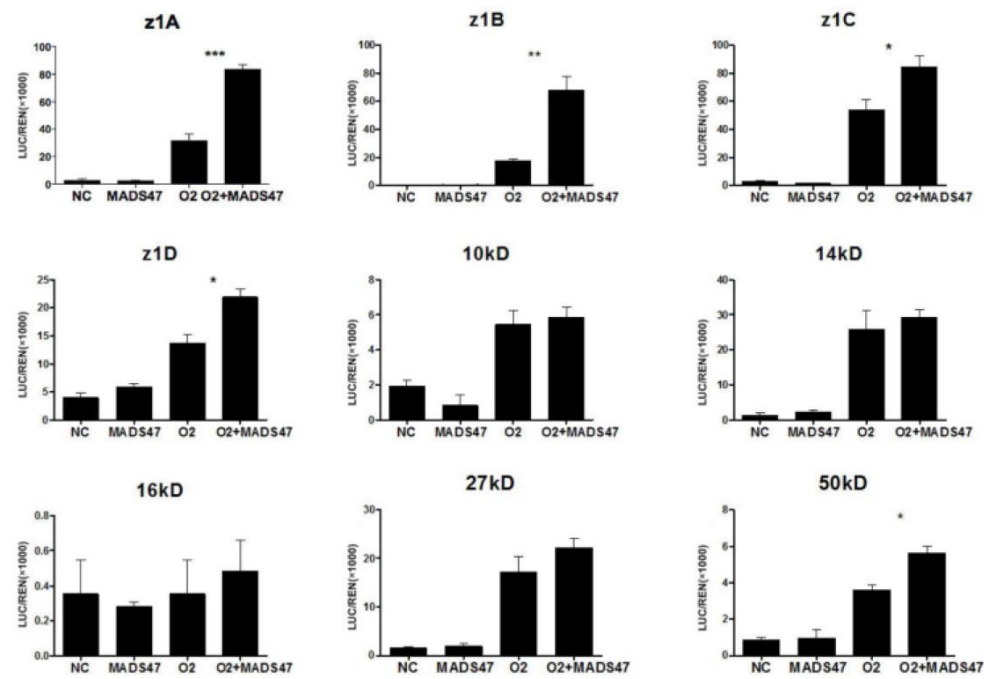

Supplement: S8 Fig — LUC/REN is the ratio of luciferase activity and reniformis activity. Error bars represent SD (n = 6) (*P < 0.05, **P < 0.01, ***P < 0.001, Student’s t test). y-axis represents the ratio of LUC/REN. (PDF) [file pgen.1005991.s008.pdf]

**S9 Fig**

## **GST Pull-down**

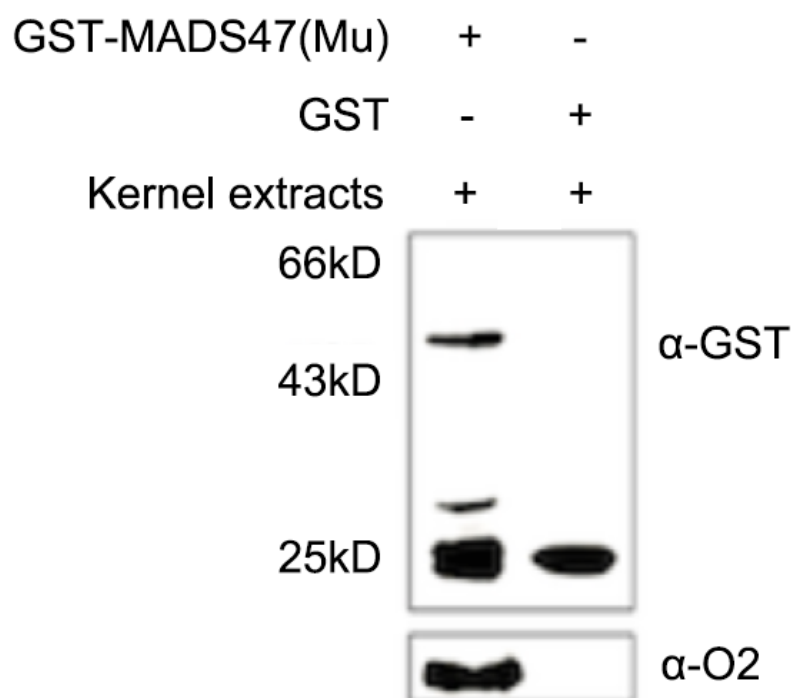

Supplement: S9 Fig — GST antibody and Opaque2 antibody were used to detect ZmMADS47(Mu)-GST fusion protein and Opaque2, respectively. (PDF) [file pgen.1005991.s009.pdf]

S10 Fig

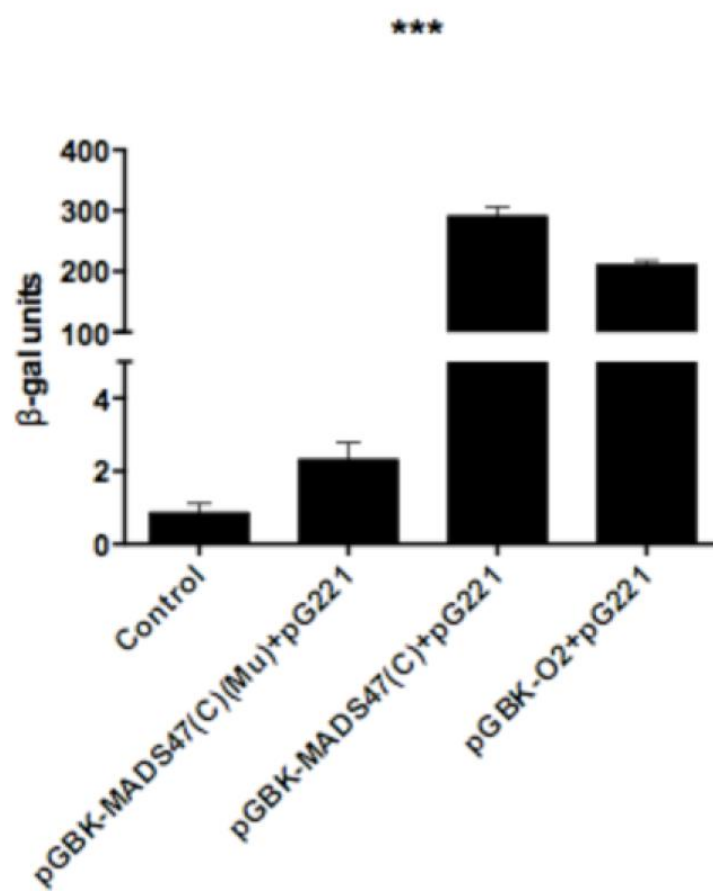

Supplement: S10 Fig — Error bars represent SD (n = 3) (***P<0.001, Student’s t test). (PDF) [file pgen.1005991.s010.pdf]
