# Supplementary material for: ZmMADS47 Regulates Zein Gene Transcription through Interaction with Opaque2
Source: PLoS Genet. 2016 Apr 14;12(4):e1005991. doi: 10.1371/journal.pgen.1005991 (PMC4831773; doi:10.1371/journal.pgen.1005991)
Supplement: S7 Fig — The blue letters represent TATA box in z1A α-zein promoter and 50-kD γ-zein promoter. (PDF) [file pgen.1005991.s007.pdf]

S7 Fig

Z1A promoter

gaatcgtgccatgatttttttctagtggaaaatagccaaaccaagcaacacatatgtg

O2 binding site (Z1) MADS binding site (Z2)

gctatcgttacacatgtgtaaaggtattgcatcacaccattgtcacccatgtatttgg

acaataccgagaggaaaaaccacttatttattgtattatatcaagtttgtcttgctta

cgtataaattataacccaacaaagtaatcactaaatgtcaaaaccaactagataccat

gtcatctctaccttatcttactaatattctttttgcaaaatccaaaattaatcttgca

caagcacaaggactgagatgtgtataaatatctcttaaattagtagctaatatatcgc

acatattattgagaccaactagcaacatagaaagcacaatagtgtaccaaca

50kD promoter

MADS47 binding site

tgagagcaatggtatgcaaatcctttgcatgtacgcaaaactagctagttgtcacaag

ttgtatatcgattcgtcgcgtttcaacaactcatgcaacattacaaacaagtaacaca

O2 binding site

atattacaaagttagtttcatacaaagcaagaaaaggacaataatacttgacatgtaa

agtgaagcttattatacttcctaatccaacacaaaacaaaaaaaagttgcacaaaggt

ccaaaaatccacatcaaccattaacctatacgtaaagtgagtgatgagtcacattatc

caacaaatgtttatcaatgtggtatcatacaagcattgacatcccataaatgcaagaa

attgtgccaacaaagctataagtaaccctcatatgtatttgcactcatgcatcacaaa

acatccttctatcagtaccatcaatcatcattcatcttagtagtataggcaccaaatc

aaatctgcaacatcaattatctaactccaaaaacca
