# Supplementary material for: ZmMADS47 Regulates Zein Gene Transcription through Interaction with Opaque2
Source: PLoS Genet. 2016 Apr 14;12(4):e1005991. doi: 10.1371/journal.pgen.1005991 (PMC4831773; doi:10.1371/journal.pgen.1005991)
Supplement: S1 Table — (PDF) [file pgen.1005991.s011.pdf]

S1 Table. Gene ontology classifications of DEGs with functional annotation in Zm*MADS47* RNAi transgenic line.

	Gene ID	Annotation	Fold-change	P-value
GO:0009611	GRMZM2G423898	unknown	1.51	0.048
response to wounding	AC199487.4_FG003	unknown	4115226.33	4.04E-13
P-value：0.00392	GRMZM2G024827	unknown	1314060.45	0.00029
	GRMZM2G012806	subtilisin-chymotrypsin inhibitor CI-1B	-1.74	1.84E-38
	AC209765.3_FG001	unknown	1364256.48	0.00029
	GRMZM2G012928	unknown	-2.96	2.96E-171
GO:0009652	GRMZM2G368861	Defensin-like protein 2 (Gamma-zeathionin-2)	-1.84	1.63E-10
defense response	GRMZM2G115340	unknown	-1.81	0.0045
P-value：0.00213	GRMZM2G368890	Defensin-like protein 1 (Gamma-zeathionin-1)	-1.5	5.75E-07
	GRMZM2G179248	unknown	-1.74	0.0077
	AC196417.3_FG008	unknown	1.49	0.0020
	AC208126.3_FG001	gamma-thionins family protein	-1.72	0.012

	GRMZM2G419675	unknown	-5.34	0.00029
GO:0044262	GRMZM2G443833	unknown	2.5	0
cellular carbohydrate metabolic process	GRMZM2G007404	unknown	2.08	0.0072
P-value：0.00392	GRMZM2G102183	Malate synthase, glyoxysomal (EC 2.3.3.9)	-2.51	0.0017
	GRMZM2G068506	unknown	1.54	1.52E-24
	GRMZM2G071630	Glyceraldehyde-3-phosphate dehydrogenase, cytosolic 3	1.47	1.01E-05
	GRMZM2G044027	unknown	1.52	0.028
	GRMZM2G026980	xyloglucan endotransglycosylase homolog1	-2.63	0.024
	GRMZM2G064302	Enolase 1 (2-phosphoglycerate dehydratase 1)	1.49	6.38E-09
	GRMZM2G176307	cytosolic glyceroldehyde-3-phosphate dehydrogenase GAPC4	1.54	6.02E-15
	GRMZM2G141399	starch synthase DULL1	1.49	0.0032
	GRMZM2G119175	Glycoside hydrolase, subgroup, catalytic core	1.43	0.012

	GRMZM2G089713	Sucrose synthase 1 (Shrunken-1)	1.52	5.18E-12
	GRMZM2G406945	unknown	7.14	0.030
	GRMZM2G018820	unknown	-1.45	0.0040
GO:0045735	GRMZM2G045387	Zein seed storage protein	-1.77	2.94E-71
Description: nutrient reservoir activity	GRMZM2G088365	Zein seed storage protein	-4.47	0
P-value:1.37E-20	GRMZM2G160739	Zein seed storage protein	-1.51	7.38E-56
	GRMZM2G060429	Bifunctional inhibitor/plant lipid transfer protein	1.41	2.34E-11
	GRMZM2G044152	Zein seed storage protein	-1.6	7.68E-72
	AF546187.1_FG007	Zein seed storage protein	-1.84	1.51E-112
	GRMZM2G388461	Zein seed storage protein	-1.7	1.14E-08
	AF546188.1_FG003	Zein seed storage protein	-1.7	0.0043
	GRMZM2G026703	RmlC-like jelly roll fold;Cupin 1	-1.89	5.89E-35

GRMZM2G346897	Zein seed storage protein	-1.89	0
GRMZM2G346895	Zein seed storage protein	-1.71	9.01E-29
GRMZM2G397687	Zein seed storage protein	-1.52	0
GRMZM2G067919	RmlC-like jelly roll fold;Cupin 1	-1.83	2.27E-21
AF546187.1_FG001	Zein seed storage protein	-2.06	3.55E-193
GRMZM2G088273	Zein seed storage protein	-2.71	0.00090
GRMZM2G008913	Zein seed storage protein	-1.71	0.0018
AF546188.1_FG007	Zein seed storage protein	-1.55	0
GRMZM2G089493	hypothetical protein LOC100191708	-4	0.020
GRMZM2G008341	Zein seed storage protein	1.64	0.027
GRMZM2G044625	Zein-alpha PZ22.3 Precursor (22 kDa zein PZ22.3)	-1.48	3.16E-304

	GRMZM2G044152	Zein-alpha ZA1/M1 Precursor (22 kDa zein ZA1/M1)	-1.6	7.68E-72
	GRMZM2G138689	unknown	-1.44	1.20E-133
	GRMZM2G332259	Rapid ALkalinization Factor	4.35	0.0098
GO:0004867	GRMZM2G423898	Proteinase inhibitor I13, potato inhibitor I	1.52	0.048
Description: serine-type endopeptidase inhibitor activity	GRMZM2G156632	Proteinase inhibitor I12, Bowman-Birk	-1.9	0.00000025
P-value：0.000948	AC199487.4_FG003	Proteinase inhibitor I13, potato inhibitor I	4115226.34	4.04E-13
	GRMZM2G024827	Proteinase inhibitor I13, potato inhibitor I	1314060.45	0.00029
	GRMZM2G012806	Proteinase inhibitor I13, potato inhibitor I	-1.74	1.84E-38
	AC209765.3_FG001	Proteinase inhibitor I13, potato inhibitor I	1364256.48	0.00029
	GRMZM2G012928	Proteinase inhibitor I13, potato inhibitor I	-2.96	2.96E-171
