# Supplementary material for: ZmMADS47 Regulates Zein Gene Transcription through Interaction with Opaque2
Source: PLoS Genet. 2016 Apr 14;12(4):e1005991. doi: 10.1371/journal.pgen.1005991 (PMC4831773; doi:10.1371/journal.pgen.1005991)
Supplement: S2 Table — (PDF) [file pgen.1005991.s012.pdf]

S2 Table. Probes used for EMSA.

z1A-8	atattattgagaccaactagcaacatagaaagcacaatagtgtaccaaca
z1A-7	gactgagatgtgtataaatatctcttaaattagtagctaatatatcgcac
z1A-6	tactaatattctttttgcaaaatccaaaattaatcttgcacaagcacaag
z1A-5	atcactaaatgtcaaaaccaactagataccatgtcatctctaccttatct
z1A-4	attatatcaagtttgtcttgcttacgtataaattataacccaacaaagta
z1A-3	tcacccatgtatttggacaataccgagaggaaaaaccacttatttattgt
z1A-2	catatgtggctatcgttacacatgtgtaaaggtattgcatcacaccattg
z1A-1	gaatcgtgccatgattttttctagtggaaaatagccaaaccaagcaaca
19kD-Z1	tctttaaaccgattattacacaagttaaccacactaaaattaacaatggt
19kD-Z2	gaatcgtgccatgattttttctagtggaaaatagccaaaccaagcaaca
50kD-1	gcaaatcctttgcatgtacgcaaaactagctagttgtcacaagttgtata
50kD-2	agcaagaaaaggacaataatacttgacatgtaaagtgaagcttattatacttccta
Z2-Mu1	cacaccattgttgcccatgtatttggacaa
Z2-Mu2	cacaccattgtcattcatgtatttggacaa
Z2-Mu3	cacaccattgtcacctgtgtatttggacaa
Z2-Mu4	cacaccattgtcacccacatatttggacaa
Z2-Mu5	cacaccattgtcacccatgcgtttggacaa
Z2-Mu6	cacaccattgtcacccatgtacctggacaa
Z2-Mu7	cacaccattgtcacccatgtatccggacaa
19kD 6-1	gaaaaaccacttatttattgt
19kD 6-2	tcacccatgtatttggacaataccgagag
19kD 7-1	ggtattgcatcacaccattg
19kD 7-2	catatgtggctatcgttacacatgtgtaaa
