# Supplementary material for: ZmMADS47 Regulates Zein Gene Transcription through Interaction with Opaque2
Source: PLoS Genet. 2016 Apr 14;12(4):e1005991. doi: 10.1371/journal.pgen.1005991 (PMC4831773; doi:10.1371/journal.pgen.1005991)
Supplement: S3 Table — (PDF) [file pgen.1005991.s013.pdf]

S3 Table. Primers involved in this paper.

**Yeast two hybrid interaction**	BD-ZmMADS47	CGGAATTCATGGTCGGGACCGGGAAGA
		CGGGATCCTCACTTGGAGCTGAAGAGTGGTA
	AD-O2(bZIP)	GGGAATTCCATATGGTGGTGCCGAACTCTTGTTG
		TCCCCCGGGGTCGTTGTACTTCTGGTTCA
**Pull-down and antibody preparation**	His-O2	CGCCATGGATGGAGCACGTCATCTCAATG
		CGGTCGACATACATGTCCATGTGTATGGC
	GST-ZmMADS47	CGGAATTCATGGTCGGGACCGGGAAGA
		CGGCGGCCGCTCACTTGGAGCTGAAGAGTGGTA
**Sub-cellular localization**	CFP-ZmMADS47(Fl)	CGGAATTCATGGTCGGGACCGGGAAGA
		CGCCCGGGTCACTTGGAGCTGAAGAGTGGTA
	CFP-ZmMADS47(C)	GCGAATTCAAACACGGATGTCGAGGATGGA
		GCCCCGGGTCACTTGGAGCTGAAGAGTG
**Transactivation testing in yeast**	BD-ZmMADS47(N)	GCCCATGGAAATGGTCGGGACCGGGAAGAG
		GCGAATTCCTTGGAATGAGAGTCGTACC
	BD-ZmMADS47(K)	GCCCATGGAATACGACTCTCATTCCAAGAC
		GCGAATTCCACTTGCTCCTTCAGCCTTG
	BD-ZmMADS47(C)	GCCCATGGAAACACGGATGTCGAGGATGGA
		GCGAATTCTCACTTGGAGCTGAAGAGTG
**RNAi line construction**	ZmMADS47(RNAi)	GCGGATCCATTTAAATAGCAGGTAATCGATCGGTAC
		GCTCTAGACCATGGTCGATCAGCTGCGTTCTCTT
**EMSA**	His-ZmMADS47	CGCCATGGATGGTCGGGACCGGGAAGA
		CGGTCGACTCACTTGGAGCTGAAGAGTGGTA
**Transactivation testing in onion epidermal cells**	35S:O2	CGCCCGGGATGGAGCACGTCATCTCAATG
		CGGAATTCATACATGTCCATGTGTATGGC
	35S:ZmMADS47	CGCCCGGGATGGTCGGGACCGGGAAGA
		CGGAATTCTCACTTGGAGCTGAAGAGTGGTA
	P19Kd(z1A)	GCAAGCTTACTTCTAAGAATTTGGTATGCCAGT

		GCGCGGCCGCTCACTCGATCCCCACCGATA
	P19Kd(z1B)	GCAAGCTTTTCTAGGGTACCAGGCCTCC
		GCGCGGCCGCTGCAGAAAGAGCAAGGAGCA
	P22Kd(z1C)	GCAAGCTTTCCGGTATGCACTGAAAGCA
		GCGCGGCCGCGCTCAAAATATTATGGGTGATGGTT
	P19Kd(z1D)	GCAAGCTTATCAACTCTTGTTCATCATT
		GCGCGGCCGCTGGTGCTAAGATGTTGCTAG
	P14kd	GCAAGCTTTGCTGCCCTGCTGGAATAAA
		GCGCGGCCGCTAACGCTTAGACCACTGGAAGC
	P27kd	GCCTCGAGTCTCTGTGTGCAAAGAAACA
		GCGCGGCCGCCGGGTTCTTCTGCGCTCTGG
	P50Kd	GCGTCGACTTGAGAGCAATGGTATGCAAATCCT
		CGGGATCCGGTTTTTGGAGTTAGATAATTGATG
	P10Kd	GCGTCGACCCGTATAAGAGGGCTAAGATCTAAT
		CGGGATCCGGCGGTGGTGTCCTTGCTTCCTAGA
	35S:O2(Mu)	GCCCCGGGATGACACCGCCGCCGGTGGTGGTGGTG
		GCGAATTCCTAATACATGTCCATGTGTATGGCC
	35S:ZmMADS47(Mu)	CGCCCGGGATGGTCGGGACCGGGAAGA
		GCGAATTCAGTCCCGAGCCTGAGCGATGTGT
